# Longitudinal blood pressure trajectories and time to stroke among hypertensive patients: A Bayesian multivariate joint modeling study in Benishangul-Gumuz, Ethiopia

**DOI:** 10.1371/journal.pone.0359435

**Published:** 2026-10-23

**Authors:** Solomon Keflie Assefa, Kebebe Tadesse, Sani Yenus, Tewodros Tegene, Solomon Debela Bekeko, Helina Mebratu Abegaz, Simachew Getaneh Endalemew, Dinkinshe Beyene Feleke, Asfaw Bessie Bemgako, Abiyu Eshetu Gobena, Bruktaut Addisu Redi, Dejen Kahsay Asgedom

**Affiliations:** 1 Department of Public Health, Pawi Health Science College, Pawi, Ethiopia; 2 Department of Medical Laboratory Sciences, College of Medicine and Health Sciences, Dire Dawa University, Dire Dawa, Ethiopia; 3 Department of Nursing, Pawi Health Science College, Pawi, Ethiopia; 4 Department of Epidemiology and Biostatistics, Institute of Public Health, College of Medicine and Health Sciences, University of Gondar, Gondar, Ethiopia; 5 Department of Veterinary Science, School of Veterinary Medicine, Bahir Dar University, Bahir Dar, Ethiopia; 6 Benishangul Gumuz Regional health bureaus, Assosa, Ethiopia; 7 Department of Medicine, School of Medicine, College of Medicine and Health Science, University of Gondar, Gondar, Ethiopia; 8 Department of Public Health, College of Medicine and Health Science, Samara University, Samara, Ethiopia; University of Montenegro-Faculty of Medicine, MONTENEGRO

## Abstract

**Background:**

Stroke is a major cause of morbidity and mortality worldwide, with hypertension being its most important modifiable risk factor. Although the association between elevated blood pressure and stroke is well established, the extent to which longitudinal blood pressure patterns influence the timing of stroke remains poorly understood, particularly in low-resource settings. We investigated the association between longitudinal systolic and diastolic blood pressure trajectories and time to stroke among hypertensive patients in Ethiopia.

**Methods:**

We conducted a retrospective cohort study of 494 newly diagnosed hypertensive patients receiving follow-up care at two general hospitals in Benishangul-Gumuz, Ethiopia, between January 1, 2015, and September 1, 2024. Time to stroke was analyzed using a Cox proportional hazards model, while repeated systolic and diastolic blood pressure measurements were modelled using linear mixed-effects models. The longitudinal and survival processes were jointly estimated using a Bayesian multivariate joint modelling framework. Posterior adjusted hazard ratios (AHRs) with 95% credible intervals (CrIs) were estimated.

**Results:**

The incidence of stroke was 2.19 per 100 person-years (95% CI: 1.60–2.64). Higher current underlying systolic and diastolic blood pressure levels were associated with increased stroke risk, with posterior AHRs of 3.25 (95% CrI: 1.85–5.83) and 2.32 (95% CrI: 1.35–4.27), respectively. Older age (≥65 years; AHR: 6.18; 95% CrI: 2.32–18.05), comorbidity (AHR: 4.00; 95% CrI: 1.72–10.25), and stage II hypertension (AHR: 4.06; 95% CrI: 1.49–16.36) were associated with earlier stroke occurrence. Rural residence was associated with a lower hazard of stroke (AHR: 0.16; 95% CrI: 0.05–0.40).

**Conclusions:**

Higher longitudinal systolic and diastolic blood pressure trajectories were associated with earlier stroke occurrence. These findings support regular blood pressure monitoring and targeted prevention strategies among older adults and patients with comorbidities in similar resource-limited settings.

## Introduction

Stroke remains one of the leading causes of death and disability worldwide, with hypertension as its most important and preventable risk factor [1–3]. Globally, an estimated 1.28 billion adults aged 30–79 years live with hypertension, and nearly two-thirds reside in low- and middle-income countries (LMICs) [4]. The African Region bears the highest prevalence at 27% [3]. Between 1975 and 2015, the number of adults with hypertension more than doubled from 594 million to 1.13 billion driven largely by increases in LMICs, particularly in Sub-Saharan Africa [5]. In sub-Saharan Africa, an estimated 74.7 million adults were living with hypertension, with earlier projections suggesting that this burden would continue to increase substantially in the absence of effective prevention and control strategies [6]. In Ethiopia, hypertension has become a major public health concern, with a pooled prevalence of 21.81%, although considerable regional variation exists, including a prevalence of 14.5% in Benishangul-Gumuz [7,8].

Hypertension, defined as persistent systolic blood pressure (SBP) ≥140 mmHg and/or diastolic blood pressure (DBP) ≥90 mmHg measured on repeated clinical assessments [9], is frequently asymptomatic but progressively damages the cardiovascular system and substantially increases the risk of stroke. Stroke, characterized by an acute vascular injury to the central nervous system, is broadly classified into ischemic and hemorrhagic subtypes [10–12]. Elevated blood pressure is responsible for a substantial proportion of stroke-related morbidity and mortality worldwide. Individuals with uncontrolled hypertension have up to a sevenfold higher risk of stroke, and approximately 70% of patients with intracerebral hemorrhage have coexisting hypertension [3,13,14].

Stroke risk among hypertensive patients is determined by a complex interplay of non-modifiable factors, including age, sex, and genetic susceptibility, and modifiable factors such as diabetes mellitus, poor adherence to antihypertensive treatment, unhealthy diet, tobacco use, harmful alcohol consumption, physical inactivity, obesity, and dyslipidemia [15–17]. Nevertheless, hypertension remains the single most important preventable determinant of stroke globally and in Ethiopia [1,2]. Beyond increasing stroke occurrence, inadequate blood pressure control accelerates disease progression, resulting in earlier stroke onset [18,19]. Previous studies have identified older age, urban residence, advanced hypertension stage, obesity, alcohol consumption, medication non-adherence, and comorbid conditions as important predictors of earlier stroke among hypertensive patients [1,20–22].

Despite the growing burden of hypertension and stroke, evidence from Ethiopia remains methodologically limited. Most studies have been cross-sectional or have relied on conventional regression methods that do not adequately account for repeated blood pressure measurements, within-patient correlation, measurement error, or individual heterogeneity over time [1,2,23]. Even longitudinal studies have typically analyzed blood pressure trajectories and stroke outcomes separately, thereby overlooking their inherent dependence and potentially producing biased or inefficient estimates [1,24]. This limitation is important because longitudinal patterns of blood pressure provide substantially richer prognostic information than single baseline measurements and may improve prediction of stroke risk [25].

To address these gaps, we investigated the association between longitudinal systolic and diastolic blood pressure trajectories and time to stroke among hypertensive patients in Benishangul-Gumuz, Ethiopia. We employed a Bayesian multivariate joint modelling framework that simultaneously analyses repeated blood pressure measurements and time-to-stroke outcomes within a unified model, thereby accounting for measurement error, within-patient correlation, and informative dropout while improving estimation efficiency [25–27]. By characterizing the dynamic relationship between blood pressure trajectories and stroke risk, this study provides robust evidence to support risk stratification and inform targeted prevention strategies for hypertensive patients in resource-limited settings.

## Methods and materials

### Study setting and period

The study was conducted in the Benishangul-Gumuz Regional State, located in western Ethiopia. According to the 2022 population estimate, the region has approximately 1.22 million inhabitants, with the majority (86.5%) residing in rural areas and 13.5% living in urban settings [28]. Healthcare services in the region are provided through a network of primary healthcare facilities and referral hospitals.

The study was conducted at two general hospitals: Assosa General Hospital and Pawi General Hospital. Assosa General Hospital, located in Assosa town, the regional capital, serves as the major referral center for patients from surrounding districts. Pawi General Hospital is located in Pawi district in the northwestern part of the region and provides essential clinical services to the northern part of the region.

A retrospective review of medical records was conducted for hypertensive patients who received follow-up care between January 1, 2015, and December 30, 2024. Data extraction was performed from April 1 to May 10, 2025.

### Study design and data sources

A retrospective institutional-based follow up study was conducted among adult hypertensive patients receiving follow-up care at Assosa and Pawi General Hospitals in Benishangul-Gumuz, Ethiopia. The study used routinely collected clinical data extracted from patient medical records and hypertension follow-up registries. Data included socio-demographic characteristics, clinical measurements, treatment history, comorbid conditions, and repeated blood pressure measurements recorded during follow-up visits. These longitudinal clinical records enabled assessment of individual blood pressure trajectories and their association with time to stroke occurrence.

### Study population and eligibility criteria

The source population comprised all adult hypertensive patients receiving follow-up care at Assosa and Pawi General Hospitals in Benishangul-Gumuz, Ethiopia. The study population included newly diagnosed hypertensive patients aged 18 years or older who initiated follow-up care between January 1, 2015, and September 1, 2024, and had at least two documented follow-up visits with recorded blood pressure measurements. Patients were excluded if their medical records lacked a documented date of hypertension diagnosis, if they had a history of stroke before the initiation of hypertension follow-up, or if the date of stroke occurrence during the follow-up period could not be determined from the available records.

### Sample size determination

The sample size was determined separately for the two components of the study: the longitudinal blood pressure trajectory analysis and the time-to-stroke analysis. For the longitudinal component, the sample size was calculated based on a two-sided significance level of 5% (α = 0.05), 80% statistical power, 15 repeated measurements per participant ((J = 15)), an assumed within-subject correlation coefficient (ρ= 0.5), a Cohen’s effect size of 0.8, and previously reported variance estimates of 15.0348 for systolic blood pressure (SBP) and 9.5268 for diastolic blood pressure (DBP) from a study conducted at Felege Hiwot Referral Hospital, Ethiopia [29]. Based on these assumptions, the required sample sizes were estimated to be 393 participants for SBP and 249 participants for DBP.

For the survival component, the sample size was calculated using the method described by Schoenfeld [30]. assuming a two-sided significance level of 5%, 80% power, an estimated stroke incidence proportion of 18.8% among hypertensive patients ((p = 0.188)), and an expected hazard ratio of 1.88 for patients with comorbidities based on previous evidence from Felege Hiwot Referral Hospital, Ethiopia [1]. The required sample size for detecting the expected association with time to stroke was estimated to be 482 participants.

Since the survival component required the largest sample size, it was used as the basis for determining the final study sample. To account for potential incomplete medical records and missing information, a 10% contingency was added, resulting in a final required sample size of 530 participants. After reviewing available records, 494 eligible patients with complete information were included in the final analysis.

### Sampling procedure

A sampling frame comprising 3,120 newly diagnosed hypertensive patients who initiated follow-up care between January 1, 2015, and September 1, 2024, was established using unique medical record numbers obtained from the hypertension registration books of Assosa and Pawi General Hospitals. From this sampling frame, 530 patients were selected using a simple random sampling technique. Following a review of the selected medical records, 36 charts were excluded because they did not meet the eligibility criteria, including missing or incomplete information on the dates of hypertension or stroke diagnosis, or documentation of stroke prior to initiation of hypertension follow-up. Consequently, 494 eligible patient records were included in the final analysis (Fig 1).

**Fig 1:**
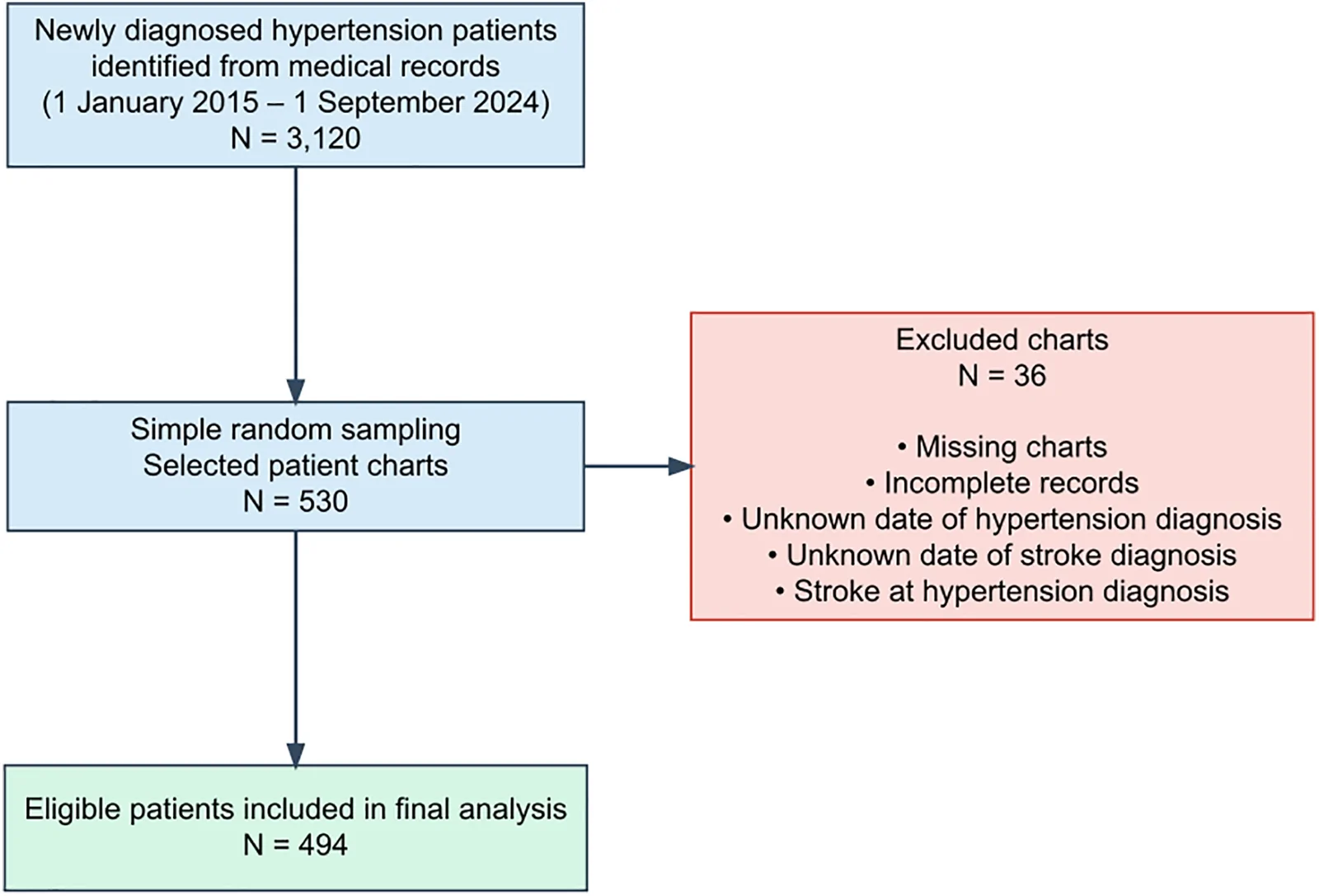
Flowchart of participant selection and eligibility for the retrospective cohort of hypertensive patients receiving antihypertensive treatment at Assosa and Pawi General Hospitals, Benishangul-Gumuz Region, Ethiopia, 2015–2024.

### Study Variables and operational definitions

For the longitudinal sub-model, the dependent variables were systolic blood pressure (SBP) and diastolic blood pressure (DBP), both measured in millimeters of mercury (mmHg). Blood pressure measurements were recorded at baseline and subsequently obtained during routine follow-up visits, which were scheduled approximately every three months. For the survival sub-model, the outcome variable was time to stroke, defined as the time from the date of hypertension diagnosis to the first documented diagnosis of stroke during follow-up, measured in months. Patients who did not experience a stroke during follow-up or were lost to follow-up were treated as censored observations.

The independent variables included sociodemographic, clinical, treatment-related, and comorbidity characteristics. Sociodemographic variables comprised age, sex, and place of residence. Clinical and treatment-related variables included hypertension stage, treatment regimen (monotherapy or combination therapy), anticoagulant use, total cholesterol, triglycerides, high-density lipoprotein cholesterol (HDL-C), low-density lipoprotein cholesterol (LDL-C), fasting blood sugar, blood urea nitrogen (BUN), and serum creatinine. Comorbid conditions included diabetes mellitus, chronic kidney disease, and coronary heart disease.

### Operational definitions

**Time to Stroke:** The primary survival outcome was defined as the time, measured in months, from the date of hypertension diagnosis to the first documented diagnosis of stroke during follow-up. Stroke was considered to have occurred if it was documented by the treating physician in the patient’s medical record. Patients who remained free of stroke until the end of follow-up, were lost to follow-up, transferred to another health facility, or died before experiencing a documented stroke event were treated as censored observations.

**Lost to Follow-up:** A patient was considered lost to follow-up if they had not attended the chronic care clinic for more than one year after their last recorded visit.

**Hypertension:** Hypertension was defined as a documented diagnosis of hypertension or the use of antihypertensive medication recorded in the patient’s medical record. Hypertension stage was classified according to the 2018 European Society of Cardiology Guidelines [31].

**Lipid profile classification:** High-density lipoprotein cholesterol (HDL-C), low-density lipoprotein cholesterol (LDL-C), total cholesterol, and triglyceride levels were classified according to the recommendations of the Mayo Clinic and the World Health Organization [32].

**Incomplete medical record:** A medical record was considered incomplete if it lacked essential information required for the analysis, including the date of hypertension diagnosis, the date of stroke diagnosis, or other key study variables.

**Time-varying covariates:** Systolic blood pressure (SBP) and diastolic blood pressure (DBP) were treated as time-varying covariates and were recorded at baseline and during routine follow-up visits, which were generally scheduled at approximately three-month intervals.

### Data collection and data quality control

Data were extracted using a structured checklist developed from patients’ medical records and informed by previous studies. Four nurses with experience in chronic outpatient care (two from each participating hospital) collected the data under the supervision of one health officer.

To ensure data quality, the extraction checklist was pretested on a sample of patient records and refined before the commencement of data collection. Data collectors received two days of training on the study objectives, eligibility criteria, and standardized data extraction procedures. Throughout the data collection period, the principal investigator and the supervisor closely monitored the process by reviewing completed checklists daily for completeness, consistency, and accuracy. Information was systematically extracted from multiple components of the medical records, including physician notes, follow-up charts, laboratory reports, medication records, and hypertension registry books to maximize data completeness and minimize extraction errors.

### Data management and analysis

Data were cleaned, coded, and analyzed using R version 4.4.1. Continuous variables were summarized using the median and interquartile range (IQR), and categorical variables using frequencies and percentages. Stroke-free survival was estimated using the Kaplan–Meier method and compared between groups using the log-rank test [33]. Univariable Cox proportional hazards regression was initially used to screen potential predictors of stroke. Variables with a p-value ≤0.25 were considered for inclusion in the multivariable analysis. The proportional hazards assumption was assessed using Schoenfeld residuals [34].

For the longitudinal analysis, exploratory plots were used to examine individual blood pressure trajectories and overall patterns over follow-up. Potential outliers were assessed using boxplots, and the distributions of systolic blood pressure (SBP) and diastolic blood pressure (DBP) were examined using histograms and quantile–quantile plots. SBP and DBP were square-root transformed before fitting the longitudinal models to improve distributional properties and stabilize variance. Estimates from the longitudinal sub-models were therefore interpreted on the square-root-transformed scale.

Linear mixed-effects models were fitted separately for SBP and DBP using restricted maximum likelihood estimation [35]. Candidate models included different random-effects structures and covariance specifications, including random-intercept, random-intercept-and-slope, and spline-based models. Models were compared using the Akaike Information Criterion (AIC) and Bayesian Information Criterion (BIC) [35].

The selected longitudinal sub-models were subsequently incorporated into a Bayesian multivariate joint modelling framework to simultaneously model the longitudinal SBP and DBP measurements and time to stroke [26]. The joint model incorporated the underlying true longitudinal blood pressure trajectories rather than the observed measurements directly and accounted for the association between the longitudinal and survival processes through shared subject-specific random effects. Different parameterizations of the association between longitudinal blood pressure and stroke risk were considered, including current-value, slope, and cumulative exposure specifications [26].

The association between the longitudinal blood pressure processes and the hazard of stroke was quantified using posterior adjusted hazard ratios (AHRs) with 95% credible intervals (CrIs). Bayesian joint models were compared using the Deviance Information Criterion (DIC) and Watanabe–Akaike Information Criterion (WAIC). Model convergence was assessed using trace plots, effective sample sizes, and the Gelman–Rubin diagnostic [36].

Missing data were evaluated using sensitivity analyses comparing complete-case analysis with multiple imputation. The consistency of the estimates across these approaches was examined to assess the potential influence of missing observations. All analyses were performed using *nlme* [37], *survival* [38], *JMBayes2* packages [36].

### Statistical model

#### Longitudinal sub-model specification.

Before fitting the joint model, linear mixed-effects models (LMMs) were used to characterize the longitudinal evolution of systolic blood pressure (SBP) and diastolic blood pressure (DBP) over follow-up [27,35]. The objective of this sub-model was to estimate each patient’s underlying true blood pressure trajectory while accounting for within-subject correlation and measurement variability.

Let $Y_{ki}(t)$ denote the observed measurement of the $k^{th}$ longitudinal outcome for patient i at time t, where k=1 represents SBP and k=2 represents DBP. The observed measurement was assumed to consist of the underlying true trajectory $m_{ki}(t)$ plus measurement error [35]:


$$\begin{array}{c} Y_{ki}(t=m_{ki}(t)+\epsilon_{ki}(t) \end{array}$$

where the true longitudinal trajectory is modeled as:


$$\begin{array}{c} m_{ki}(t)={X_{ki}}^{T}(t)\beta_{\text{k}}+{Z_{ki}}^{T}(t)b_{ki} \end{array}$$

Combining the two expressions:


$$\begin{array}{c} Y_{ki}(t={X_{ki}}^{T}(t)\beta_{\text{k}}+{Z_{ki}}^{T}(t)b_{ki}+\epsilon_{ki}(t), \end{array}$$

$$\begin{array}{c} b_{i}={\left(\;b_{\text{1}i},\;b_{\text{2}i}\right)}^{T}\sim N(\text{0},D), \end{array}$$

$$\begin{array}{c} \epsilon_{ki}(t)\sim N(\text{0},\;{\sigma^{\text{2}}}_{k}) \end{array}$$

where ${\text{X}}_{\text{ki}}(\text{t})$ and ${\text{Z}}_{\text{ki}}(\text{t})$ are design vectors for fixed and random effects, respectively; $\beta_{\text{k}}$ represents the vector of fixed-effect parameters; ${\text{b}}_{\text{ki}}$ represents subject-specific random effects; and D is the variance-covariance matrix of the random effects.

The random-effects covariance matrix D allows correlation between the SBP and DBP trajectories, thereby accounting for the biological dependence between the two longitudinal processes. Measurement errors $\varepsilon_{\text{ki}}(\text{t})$ were assumed to be normally distributed with mean zero and variance $\sigma_{\text{k}}^{\text{2}}$, independent of the random effects.

#### Survival sub-model specification.

The time-to-stroke process was modeled using a proportional hazards framework. Let $T_{i}^{*}$ denote the true event time for patient i, and let $C_{i}$ represent the censoring time. The observed event time was defined as [26]:


$$\begin{array}{c} T_{i}=min(\;{T_{i}}^{*},C_{i}) \end{array}$$

with event indicator:


$$\begin{array}{c} \delta_{i}=I\left(T_{i}^{*}\leq C_{i}\right) \end{array}$$

where $\delta_{i}=\text{1}$ indicates occurrence of stroke and $\delta_{i}=\text{0}$ indicates censoring.

The hazard function was specified as:


$$\begin{array}{c} h_{i}(t)=h_{\text{0}}(ttextrmexp\left(\gamma^{T}W_{i}\right) \end{array}$$

where $h_{\text{0}}(t)$ is the baseline hazard function, $W_{i}$ represents baseline covariates, and γ denotes the corresponding regression coefficients [34].

#### Bayesian multivariate joint model specification.

The Bayesian multivariate joint model simultaneously estimates the longitudinal SBP and DBP trajectories and their association with time to stroke. The model assumes that the longitudinal and survival processes are conditionally independent given shared patient-specific random effects [25].

The joint model is defined as [39]:


$$\begin{array}{c} \left\{ \begin{array}{c} m_{ki}(t)=X_{ki}^{T}(t)\beta_{k}+Z_{ki}^{T}(t)b_{ki},\\ h_{i}\left(t,|M_{i}(t)W_{i}\right)=h_{\text{0}}(t)\text{exp}\left[\gamma^{T}W_{i}+\sum_{k=\text{1}}^{K}\alpha_{k}m_{ki}(t)\right],\\ b_{i}\sim N\left(\text{0},D\right) \end{array}\right. \end{array}$$

where:

- $m_{ki}(t)$ represents the latent true trajectory of the $k^{th}$ blood pressure outcome;
- $M_{i}(t)=\left\{m_{\text{1}i},(s){\;m}_{\text{2}i}(s):\text{0}\leq s<t\right\}$ represents the history of the underlying SBP and DBP trajectories up to time t;
- $\alpha_{k}$ represents the association parameter quantifying the relationship between each longitudinal blood pressure trajectory and the hazard of stroke;
- $W_{i}$ contains baseline covariates with regression coefficient vector γ.

The association structure links the estimated true blood pressure trajectories, rather than the error-prone observed measurements, with the risk of stroke. This approach reduces bias due to measurement error and appropriately accounts for the correlation among repeated blood pressure measurements [25,36].

### Bayesian estimation and model inference

Parameters were estimated using Bayesian inference based on the posterior distribution obtained by combining the likelihood function with prior distributions. Weakly informative priors were specified to minimize their influence on parameter estimation. Normal priors N(0,0.001) were assigned to fixed-effect parameters ($\beta_{k}$), survival regression coefficients (γ), and association parameters ($\alpha_{k}$). An inverse-gamma prior IG(1,0.005) was assigned to variance parameters, and appropriate priors were specified for the random-effects covariance matrix [26].

The Bayesian multivariate joint models were fitted using the *JMbayes2* package in R [40]. Posterior inference was based on 65,000 Markov Chain Monte Carlo iterations, including a burn-in period of 15,000 iterations, three parallel chains, and thinning every 10 iterations. Model convergence was assessed using trace plots, effective sample size (ESS), and Gelman–Rubin diagnostic statistics [41]. A covariate was considered statistically significant when its 95% Bayesian credible interval excluded zero for regression parameters or excluded one for hazard ratios. Model adequacy for the survival component was evaluated using Cox–Snell residuals.

### Ethics approval and consent to participate

Ethical approval for this study was granted by the Institutional Review Committee of Pawi Health Science College (Ref: PHSC/R/SEC/106/2025). The committee waived the requirement for informed consent due to the use of retrospectively collected, fully anonymized patient data. Official permission to access the data was obtained from the clinical directors of all participating general hospitals. All data were handled confidentially, stored securely, and contained no personal identifiers.

## Results

### Baseline characteristics of study participants

A total of 494 hypertensive patients were included in the final analysis. Of these, 303 (61.3%) were male and 285 (57.7%) resided in rural areas. The median age of participants was 55 years (IQR: 45–60). Regarding clinical characteristics, elevated fasting blood glucose was observed among 110 participants (47.2%), while elevated blood urea nitrogen was observed among 116 (39.3%). Among participants with available lipid measurements, high triglyceride levels were identified in 25 (56.8%), elevated total cholesterol in 20 (48.8%), LDL-C ≥ 100 mg/dL in 22 (66.7%), and low HDL-C (<40 mg/dL) in 20 (50.0%).

Diabetes mellitus was documented among 102 participants (20.8%), and coronary heart disease among 8 (1.6%). Overall, 185 participants (37.4%) had at least one documented comorbidity. At baseline, 191 (38.7%) participants were classified as having stage II hypertension, and 295 (59.7%) were receiving combination antihypertensive therapy (two or more medications). The median serum creatinine level was 0.66 mg/dL (IQR: 0.51). Detailed demographic, clinical, laboratory, and treatment characteristics of study participants are presented in Table 1.

**Table 1:** Baseline demographic, clinical, laboratory, and treatment characteristics of hypertensive patients receiving follow-up care at Assosa and Pawi General Hospitals, Benishangul-Gumuz Region, Ethiopia, 2015–2024.

Variable	Categories	Stroke status		Total (%)
		Censored (%)	Event (%)	
Name of Hospital	Pawi	260 (58.82)	30 (57.69)	290 (58.7)
	Assosa	182 (41.18)	222 (42.31)	204 (41.3)
Sex	Female	158 (35.75)	33 (63.46)	191 (38.66)
	Male	284 (64.25)	19 (36.54)	303 (61.34)
Residence	Rural	271 (61.31)	14 (26.92)	285 (57.69)
	Urban	171 (38.69)	38 (73.08)	209 (42.31)
Base line FBS ^**a**^	<125	110 (53.66)	13 (46.43)	123 (52.79)
	≥125	95 (46.34)	15 (53.57)	110 (47.21)
BUN ^**b**^	<24	158 (61.72)	21 (53.85)	179 (60.68)
	≥24	98 (38.28)	18 (46.15)	116 (39.32)
Total Triglyceride ^**c**^	<150	15 (42.86)	4 (44.44)	19 (43.18)
	≥150	20 (57.14)	5 (55.56)	25 (56.82)
Total Cholesterol ^**d**^	<200	19 (54.29)	2 (33.33)	21 (51.22)
	≥200	16 (45.71)	4 (66.67)	20 (48.78)
HDL_C e	≥40	15 (45.45)	5 (71.43)	20 (50.0)
	<40	18 (54.55)	2 (28.57)	20 (50.00)
LDL_C ^e^	<100	10 (38.46)	1 (14.29)	11 (33.33)
	≥100	16 (61.54)	6 (85.71)	22 (66.67)
Diabetes Mellitus	No	344 (78.54)	44 (84.62)	388 (79.18)
	Yes	94 (21.46)	8 (15.38)	102 (20.82)
History of CHDs	No	431 (98.18)	52 (100)	483 (98.37)
	Yes	8 (1.82)	0 (0.00)	8 (1.63)
Presence of comorbidity	No	287 (64.93)	22 (42.31)	309 (62.55)
	Yes	155 (35.07)	30 (57.69)	185 (37.45)
Baseline Stage of HTN	Stage 1	151 (34.16)	13 (25.00)	164 (33.20)
	Stage 2	160 (36.2)	31 (59.62)	191 (38.66)
	Hypertensive Crisis	131 (29.64)	8 (15.38)	139 (28.14)
Treatment combinations	Single Drug	186 (42.08)	13 (25.00)	199 (40.28)
	≥Two Drugs	256 (57.92)	39 (75.00)	295 (59.72)
Age	Median (IQR)	55 (15)	55.5 (18)	55 (15)
Creatinine	Median (IQR)	0.65 (0.48)	0.76 (0.51)	0.66 (0.51)

**Note: a**:(n = 233), **b**:(n = 295), **c**:(n = 44), **d**:(n = 41), **d**:(n = 40), **CHD** = Coronary Heart Diseases, **VDs** = Vascular Diseases, **FBS** = Fasting Blood Sugar, **HDL_C** = High Density Lipid Cholesterol, **LDL_C** = Low Density Lipid Cholesterol

### Exploring longitudinal changes in systolic and diastolic blood pressure

Exploratory analyses were performed to assess individual and population-level patterns of change in systolic blood pressure (SBP) and diastolic blood pressure (DBP) over follow-up. Individual trajectory plots were generated to examine within-subject and between-subject variability in repeated blood pressure measurements. In addition, locally estimated scatterplot smoothing (LOESS) curves were fitted to visualize the overall temporal trends in mean SBP and DBP (Fig 2).

**Fig 2:**
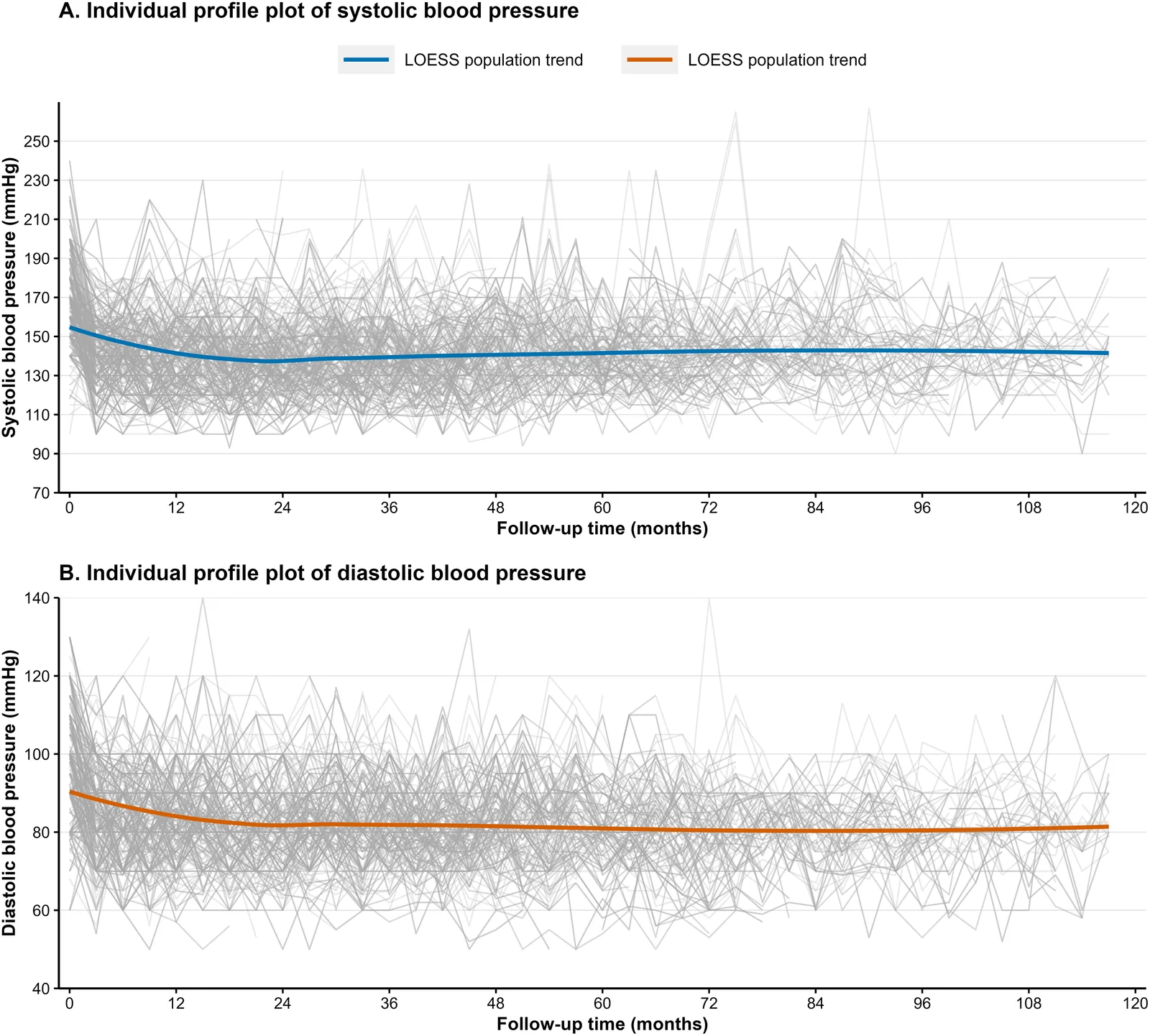
Individual longitudinal profile plots of systolic blood pressure (SBP) (A) and diastolic blood pressure (DBP) (B) among hypertensive patients receiving antihypertensive treatment at Assosa and Pawi General Hospitals, Benishangul-Gumuz Region, Ethiopia, 2015–2024.

The individual profiles demonstrated substantial heterogeneity in both baseline blood pressure levels and rates of change over time, indicating considerable variability between patients. This observed variability supported the inclusion of patient-specific random effects to account for differences in baseline blood pressure and longitudinal change patterns. The population-level LOESS curves suggested an approximately linear trend in SBP and DBP over the follow-up period, supporting the use of linear time effects in the longitudinal mixed-effects models.

Final model specifications were selected based on model fit criteria. Comparisons of linear mixed-effects models for systolic and diastolic blood pressure are presented in S1 File (Tables S3 and S4, respectively).

To further explore the longitudinal patterns of blood pressure in relation to stroke occurrence, mean profile plots were generated for patients who experienced stroke events and those who were censored during follow-up. As shown in Fig 3A and Fig 3B, the average SBP and DBP trajectories differed between the two groups. Patients who experienced stroke generally had higher mean blood pressure levels throughout follow-up compared with those who remained stroke-free. The light red and blue curves represent the mean blood pressure trajectories among patients with observed stroke events and censored observations, respectively. These exploratory findings suggest a potential association between higher longitudinal blood pressure levels and subsequent stroke occurrence, which was further evaluated using the Bayesian multivariate joint modelling approach.

**Fig 3:**
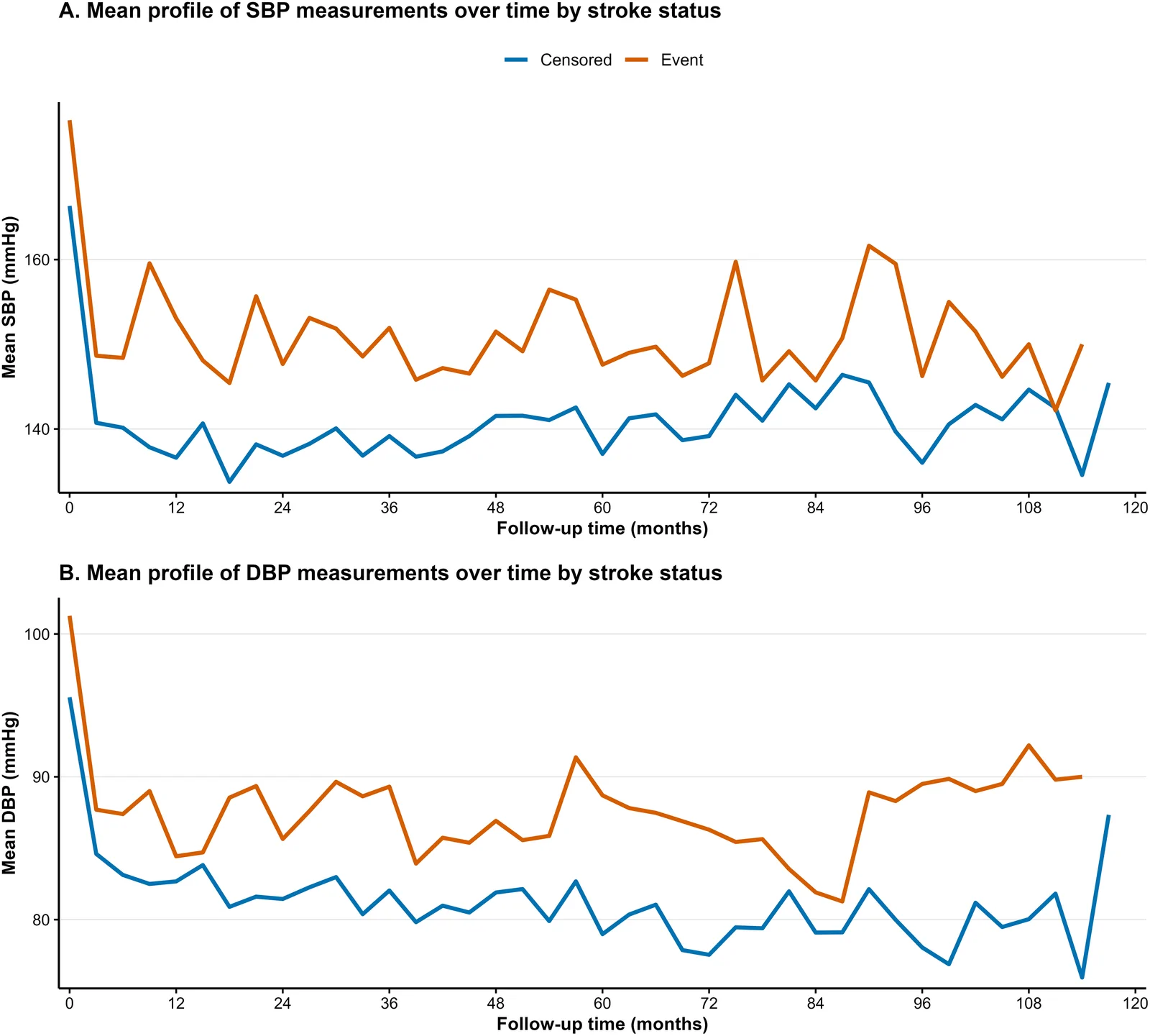
Mean longitudinal profile plots of systolic blood pressure (SBP) (A) and diastolic blood pressure (DBP) (B) according to stroke status among hypertensive patients receiving antihypertensive treatment at Assosa and Pawi General Hospitals, Benishangul-Gumuz Region, Ethiopia, 2015–2024.

### Time to Stroke among hypertensive patients

Participants were followed for a minimum of 3 months and a maximum of 117 months, with a median follow-up duration of 51 months (IQR: 56.3 months). During 28,462 person-months of observation, 52 (10.5%) participants experienced a stroke event. The overall incidence rate of stroke was 1.83 per 1,000 person-months (95% CI: 1.34–2.35), equivalent to 2.19 per 100 person-years (95% CI: 1.60–2.64).

The probability of remaining stroke-free declined progressively over the follow-up period (Fig 4). The estimated stroke-free survival probabilities at 24, 48, 72, and 96 months were 97.2%, 93.0%, 89.6%, and 83.7%, respectively. More than half of all stroke events (56.3%) occurred within the first five years of follow-up. The median stroke-free survival time was not reached, as fewer than 50% of participants experienced stroke during the observation period. The Kaplan–Meier survival curve represents the estimated stroke-free survival probability, with the shaded area indicating the corresponding 95% confidence interval (Fig 4).

**Fig 4:**
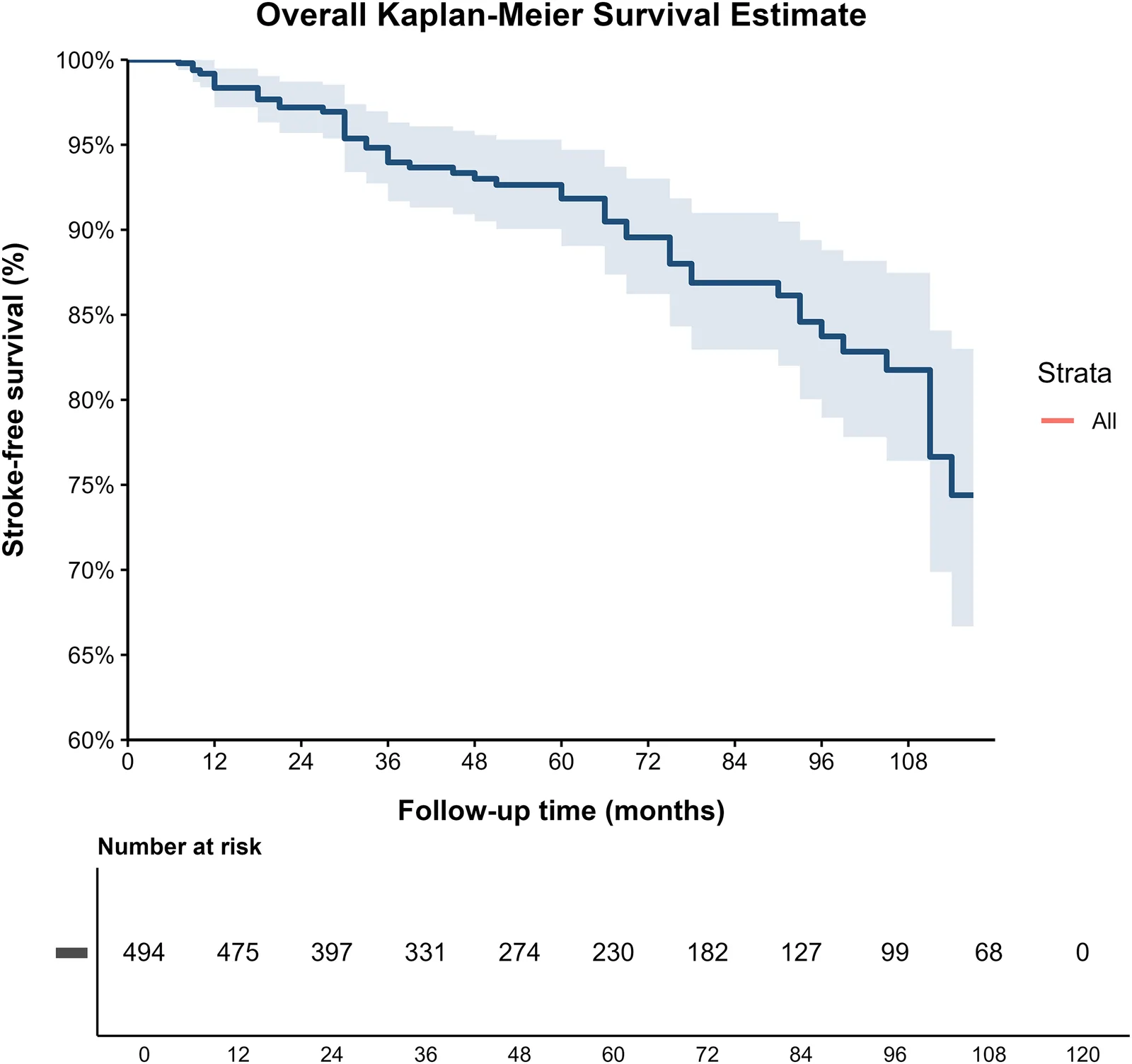
Kaplan–Meier estimate of stroke-free survival among hypertensive patients receiving antihypertensive treatment at Assosa and Pawi General Hospitals, Benishangul-Gumuz Region, Ethiopia, 2015–2024.

S1 File includes the overall Kaplan–Meier survival curve (Fig S1), stratified Kaplan–Meier curves by participant characteristics (Fig S2, plots 2–6), Cox proportional hazards regression results (Table S1), proportional hazards assumption tests (Table S2), and Cox–Snell residual diagnostics (Fig S3).

### Bayesian multivariate joint models

Candidate longitudinal and survival sub-models were evaluated separately, with model selection Criterion [26]. The selected sub-models were subsequently integrated within a Bayesian multivariate joint modelling framework estimated using Markov chain Monte Carlo (MCMC) methods.

Three association structures between the longitudinal blood pressure processes and the risk of stroke were evaluated: current value, slope, and area under the curve (AUC). Based on the Deviance Information Criterion (DIC) and Watanabe–Akaike Information Criterion (WAIC), the current-value parameterization provided the best-fitting model (Table S5 in S1 File).

MCMC diagnostics indicated satisfactory posterior sampling and convergence. The bulk and tail effective sample sizes exceeded 3,000 and 3,100, respectively, for all model parameters, and trace and posterior density plots showed satisfactory mixing (Fig S4). Detailed model building, comparisons, convergence diagnostics, and posterior diagnostic plots are provided in S1 File.

### Predictors of stroke and their association with blood pressure trajectories

In the Bayesian multivariate joint model using the current value parameterization, the estimated underlying true trajectories of systolic blood pressure (SBP) and diastolic blood pressure (DBP) were significantly associated with the hazard of stroke. A higher current value of the underlying square-root-transformed SBP and DBP trajectories was associated with an increased risk of stroke, with posterior adjusted hazard ratios (AHRs) of 3.25 (95% CrI: 1.85–5.83) and 2.32 (95% CrI: 1.35–4.27), respectively (Table 2).

**Table 2:** Bayesian multivariate joint model estimates for the survival sub-model using the current value parameterization among hypertensive patients receiving follow-up care at Assosa and Pawi General Hospitals, Benishangul-Gumuz Region, Ethiopia, 2015–2024.

Survival sub-model			
Covariates	CHR (95%CrI)	AHR (95%CrI)	Rhat
**Age**			
18-64	1	1	
≥65	5.00 (2.87-8.71)	6.18 (2.32- 18.05)	1
**Residence**			
Urban	1	1	
Rural	0.31 (0.17-0.57)	0.16 (0.05 -0.40)	
**Sex**			
Male	1	1	
Female	0.32 (0.18-0.56)	0.35 (0.10 - 1.07)	1
**Comorbidity**			
No	1	1	
Yes	2.35 (1.36 - 4.08)	4.00 (1.72- 10.25)	1
**Stage of hypertension**			
Stage I	1	1	
Stage II	2.00 (1.05- 3.83)	4.06 (1.49- 16.36)	1
Stage III	0.57 (0.24 -1.39)	0.67 (0.19- 2.42)	
**Anticoagulant use**			
No	0.33 (0.15- 0.74)	0.82 (0.24- 3.29)	
Yes	1	1	1
**Drug regimens**			
Single regimen	1	1	
≥Two regimen	0.57 (0.30- 1.06)	0.70 (0.26 - 1.89)	1
**Associations**			
**Time varying Covariates**	**Parameterizatio**n		
SBP	Current value	3.25 (1.85, 5.83)	1
DBP	Current value	2.32 (1.35, 4.27)	1

**Note**: **Rhat** = Potential Scale Reduction Factor**, ESS** = Effective Sample Size, **CrI** = Credible interval, **CHR** = Crude hazard ratio, **CI** = Confidence Interval

Several patient characteristics were also significantly associated with time to stroke. Participants aged ≥65 years had a substantially higher hazard of stroke compared with younger patients (AHR = 6.18; 95% CrI: 2.32–18.05). The presence of comorbid conditions was associated with increased stroke risk (AHR = 4.00; 95% CrI: 1.72–10.25), and patients with stage II hypertension at baseline had a higher hazard of stroke compared with those with lower hypertension stages (AHR = 4.06; 95% CrI: 1.49–16.36). Conversely, rural residence was associated with a lower hazard of stroke compared with urban residence (AHR = 0.16; 95% CrI: 0.05–0.40) (Table 2).

In the longitudinal sub-model, patients receiving combination antihypertensive therapy (two or more medications) demonstrated significant differences in their longitudinal SBP and DBP trajectories compared with those receiving fewer medications (Table 3).

**Table 3:** The Fitted Bayesian Multivariate joint model longitudinal sub-models result under current value parameterizations for patients in general hospitals of Benishangul Gumuz, Ethiopia, 2015-2024.

Longitudinal Sub-Models				
	SBP Sub Model		DBP Sub Model	
Fixed effects	Mean (95% CrI)	R hat	Mean (95% CrI)	R hat
Intercept	12.23 (12.12,	12.34)	1	1
**Visit**	−0.011(−0.014, −0.007)	1	−0.022(−0.025, −0.018)	1
**Sex**				
Female	-0.04 (-0.13, 0.05)		-0.09 (-0.16,	-0.01)
Male	1	1		1
**Age**				
18-64	1	1	1	
>65	0.21 (0.11, 0.32)	1	-0.07 (-0.15, 0.02)	1
**Comorbidity**				
No	1	1	1	
Yes	0.07 (-0.01,0.16)	1	-0.04 (-0.12, 0.03)	1
**Drug regimens**				
Single	1	1	1	
≥Two drugs	-0.29 (-0.37, -0.20)	1	-0.13 (-0.20, -0.06)	1
**Random effect components**				
Std. Dev. (Intercept)	0.52	9.54		
Std. Dev. (Residuals)	0.6971	(0.68, 0.71)	0.5126	(0.51, 0.52)

## Discussions

This study evaluated the time to stroke and its predictors among hypertensive patients receiving care in general hospitals of Benishangul-Gumuz, Ethiopia, while simultaneously examining how longitudinal changes in systolic and diastolic blood pressure influence stroke risk. Using a Bayesian multivariate joint modelling framework, we demonstrated that the underlying current levels of both systolic and diastolic blood pressure were significantly associated with stroke occurrence. In addition, older age, comorbid conditions, and stage II hypertension were important predictors of shorter stroke-free survival. These findings highlight the importance of moving beyond single blood pressure measurements toward dynamic assessment of blood pressure patterns for improved cardiovascular risk stratification.

The observed stroke incidence rate of 2.19 per 100 person-years indicates a substantial burden among hypertensive patients in this setting. This estimate is comparable with findings from hypertensive populations in other low- and middle-income countries, including pooled estimates from Asian cohorts reporting incidence rates between 1.16 and 1.83 per 100 person-years [42], and previous evidence from sub-Saharan Africa [43]. However, the incidence observed in our study was higher than that reported from Bahir Dar, Ethiopia (1.42 per 100 person-years) [1].

Differences in study setting, population characteristics, healthcare access, hypertension severity, and follow-up intensity may explain these variations. Hospital-based studies are more likely to capture patients with more advanced disease, poorer blood pressure control, or multiple cardiovascular risk factors compared with community-based studies [43,44]. Furthermore, differences in treatment availability, adherence to antihypertensive therapy, management protocols, and prevalence of comorbidities may contribute to variability in stroke incidence across settings [45,46]. In addition, methodological differences, such as how stroke was defined and diagnosed, the thoroughness of follow-up, and the duration of observation, can all affect the number of stroke events recorded and thus the reported incidence rates [45,47].

Age was one of the strongest predictors of stroke in this study, with patients aged 65 years or older experiencing a markedly increased risk compared with younger patients. This finding is consistent with previous studies demonstrating that advancing age remains one of the most important determinants of stroke among individuals with hypertension [1,48]. The increased risk among older adults may reflect cumulative vascular injury, arterial stiffness, endothelial dysfunction, and progressive atherosclerotic changes that occur with ageing [48]. In addition, impaired cerebral autoregulation among older adults may increase vulnerability to cerebrovascular injury in the presence of elevated blood pressure [49]. These findings reinforce the need for intensified cardiovascular risk assessment and more frequent blood pressure monitoring among older hypertensive patients.

Rural residence was associated with a lower hazard of stroke compared with urban counterparts. Although this finding should be interpreted cautiously given the potential influence of healthcare-seeking patterns and unmeasured socioeconomic factors, similar urban–rural disparities have been reported in several African settings. For example, stroke incidence in urban Dar es Salaam, Tanzania, was substantially higher than in rural populations [50], and comparable patterns have been observed in Egypt [51]. Several mechanisms may explain this difference, including higher prevalence of obesity, diabetes, physical inactivity, unhealthy dietary patterns, and psychosocial stressors in urban environments [45,52]. However, further research is needed to understand the contextual factors contributing to the observed lower stroke risk among rural residents, including differences in lifestyle, social support, physical activity, and healthcare utilization.

The presence of comorbid conditions was strongly associated with increased stroke risk. Patients with comorbidities had substantially higher hazard of stroke compared with those without comorbid conditions. This finding is consistent with evidence that conditions such as diabetes, chronic kidney disease, and cardiovascular diseases accelerate vascular damage, promote inflammation, and increase the likelihood of cerebrovascular events among hypertensive individuals [46,53,54]. These results emphasize that hypertension management should not occur in isolation but should incorporate integrated assessment and treatment of coexisting chronic diseases.

Baseline stage II hypertension was also associated with increased stroke risk, supporting existing evidence that the severity and duration of blood pressure elevation are important determinants of cerebrovascular outcomes [46], [55]. Persistent exposure to higher blood pressure levels contributes to endothelial dysfunction, arterial remodeling, and target-organ damage, thereby increasing susceptibility to stroke [55]. These findings highlight the importance of early identification and intensive management of patients with poorly controlled or severe hypertension.

A major contribution of this study is the demonstration that the estimated underlying current values of both SBP and DBP trajectories were significantly associated with stroke risk. Previous studies have largely relied on baseline blood pressure measurements or conventional time-dependent approaches, which may not adequately account for measurement error, within-person correlation, or the dynamic nature of blood pressure change over time [46,56,57]. By applying a Bayesian multivariate joint modelling framework, this study incorporated repeated blood pressure measurements and simultaneously modelled longitudinal blood pressure evolution and stroke occurrence. This approach provides a more clinically informative assessment of cardiovascular risk by using the patient’s underlying blood pressure trajectory rather than isolated observed measurements.

The findings suggest that routine clinical monitoring should consider longitudinal blood pressure patterns rather than relying exclusively on single clinic measurements. Incorporating repeated blood pressure information into risk prediction approaches may improve early identification of patients at increased risk of stroke, particularly among older adults, individuals with stage II hypertension, and those with multiple comorbidities. Such approaches may support more targeted prevention strategies in resource-limited settings where stroke burden is increasing.

### Strength and limitation of the study

The major strength of this study is the application of a Bayesian multivariate joint modelling framework that simultaneously evaluates longitudinal SBP and DBP trajectories and time-to-stroke outcomes. Unlike conventional approaches based on baseline measurements, this method accounts for within-patient correlation, measurement variability, and the dynamic nature of blood pressure changes over time, providing a more comprehensive understanding of the relationship between blood pressure evolution and stroke risk.

This study also has several limitations. First, the retrospective design limited the availability of important stroke risk factors, including lifestyle behaviors, medication adherence, socioeconomic characteristics, and other behavioral determinants. Second, reliance on routinely collected clinical records may have resulted in incomplete or inconsistent documentation of some variables. Third, stroke diagnosis was based on physician documentation in medical records. Because access to neuroimaging was limited in the study setting, detailed stroke classification, including imaging-confirmed diagnosis and differentiation between ischemic and hemorrhagic stroke, could not consistently be assessed. Finally, because the study was conducted in two general hospitals within a single Ethiopian region, the findings should be generalized cautiously to other populations and healthcare settings.

## Conclusions

Among hypertensive patients in Benishangul-Gumuz, Ethiopia, higher underlying current levels of systolic and diastolic blood pressure were associated with shorter time to stroke. Older age, comorbidities, and stage II hypertension were also associated with increased stroke risk. These findings support the use of repeated blood pressure measurements and longitudinal blood pressure patterns, rather than baseline measurements alone, for risk stratification. Targeted prevention and optimized hypertension management should prioritize patients with these high-risk characteristics.

## Supporting information

S1 File Supplementary analyses and model diagnostics. Detailed results of longitudinal and survival model selection, comparison of alternative Bayesian joint-model association structures, posterior convergence diagnostics, trace plots, posterior density plots, effective sample size diagnostics, and Cox–Snell residual diagnostics.
(PDF)

S1 Data Wide stroke data. (DTA)
