# Supplementary material for: Longitudinal blood pressure trajectories and time to stroke among hypertensive patients: A Bayesian multivariate joint modeling study in Benishangul-Gumuz, Ethiopia
Source: PLoS One. 2026 Oct 23;21(10):e0359435. doi: 10.1371/journal.pone.0359435 (PMC13693927; doi:10.1371/journal.pone.0359435)
Supplement: Supplementary file 1 [file pone.0359435.s001.pdf]

Supplementary File 1

Solomon Keflie Assefa

June 2025

Contents

1 Supplementary analyses **1**
1.1 Kaplan–Meier survival analysis 1
1.2 Kaplan–Meier analysis by patient characteristics 2

2 Time to stroke **9**
2.1 Cox proportional hazards regression 9
2.2 Assessment of the proportional hazards assumption 9
2.3 Cox–Snell residual diagnostics 10

3 Bayesian Multivariate Joint Modeling **11**
3.1 Bayesian Model Convergence and Diagnostics 13

1 Supplementary analyses

This supplementary file provides additional analyses supporting the main findings, including survival analyses, model building and comparison, alternative joint-model specifications, and model convergence and diagnostic assessments.

1.1 Kaplan–Meier survival analysis

Kaplan–Meier methods were used to estimate stroke-free survival over the follow-up period. The overall Kaplan–Meier curve and corresponding 95% confidence intervals are presented in Figure S1. The number of patients remaining at risk is shown below the survival curve.

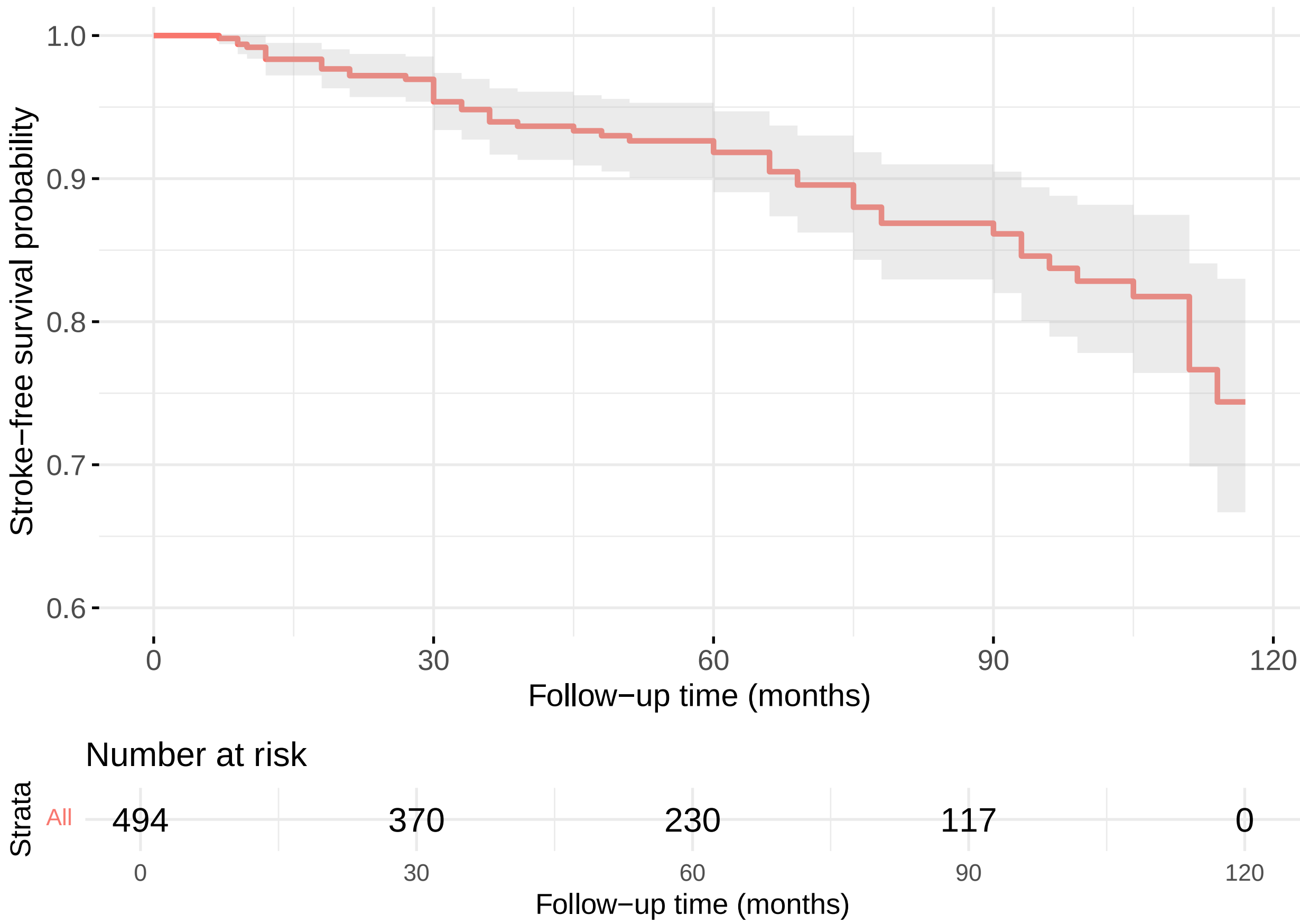

Figure S1. Kaplan–Meier estimate of stroke-free survival among hypertensive patients in general hospitals of Benishangul-Gumuz, Ethiopia, 2015–2024. Shaded areas represent 95% confidence intervals, and the risk table shows the number of patients remaining under observation at each follow-up time.

1.2 Kaplan–Meier analysis by patient characteristics

Kaplan–Meier curves were additionally examined across selected demographic, clinical, and treatment characteristics. Figure S2 presents stroke-free survival according to age group, fasting blood glucose, residence, comorbidity status, anticoagulant use, sex, and hypertension stage. Curves are presented with corresponding 95% confidence intervals.

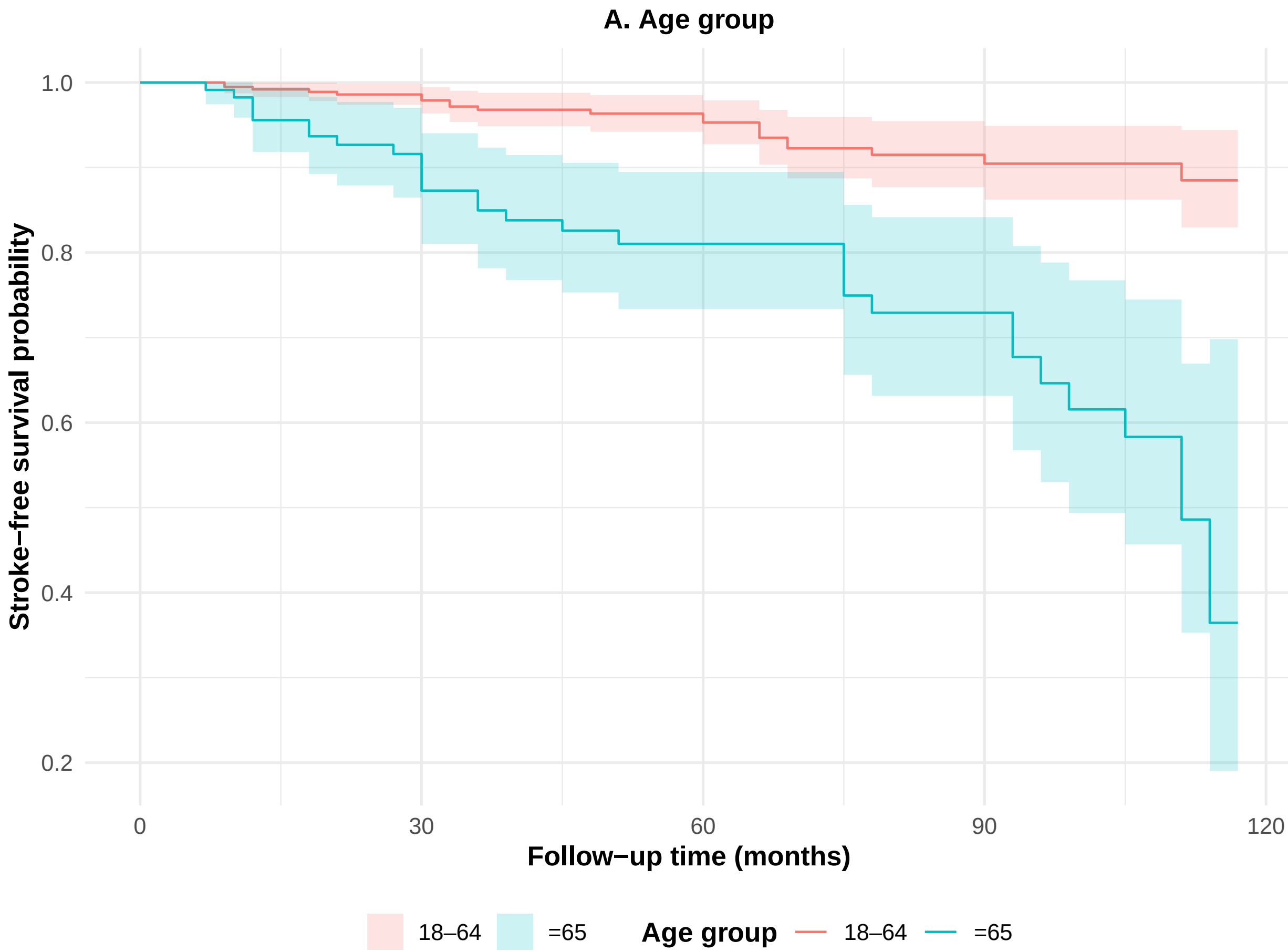
A. Age group
Stroke−free survival probability
1.0
0.8
0.6
0.4
0.2
0
30
60
90
120
Follow−up time (months)
18–64
=65
Age group
18–64
=65

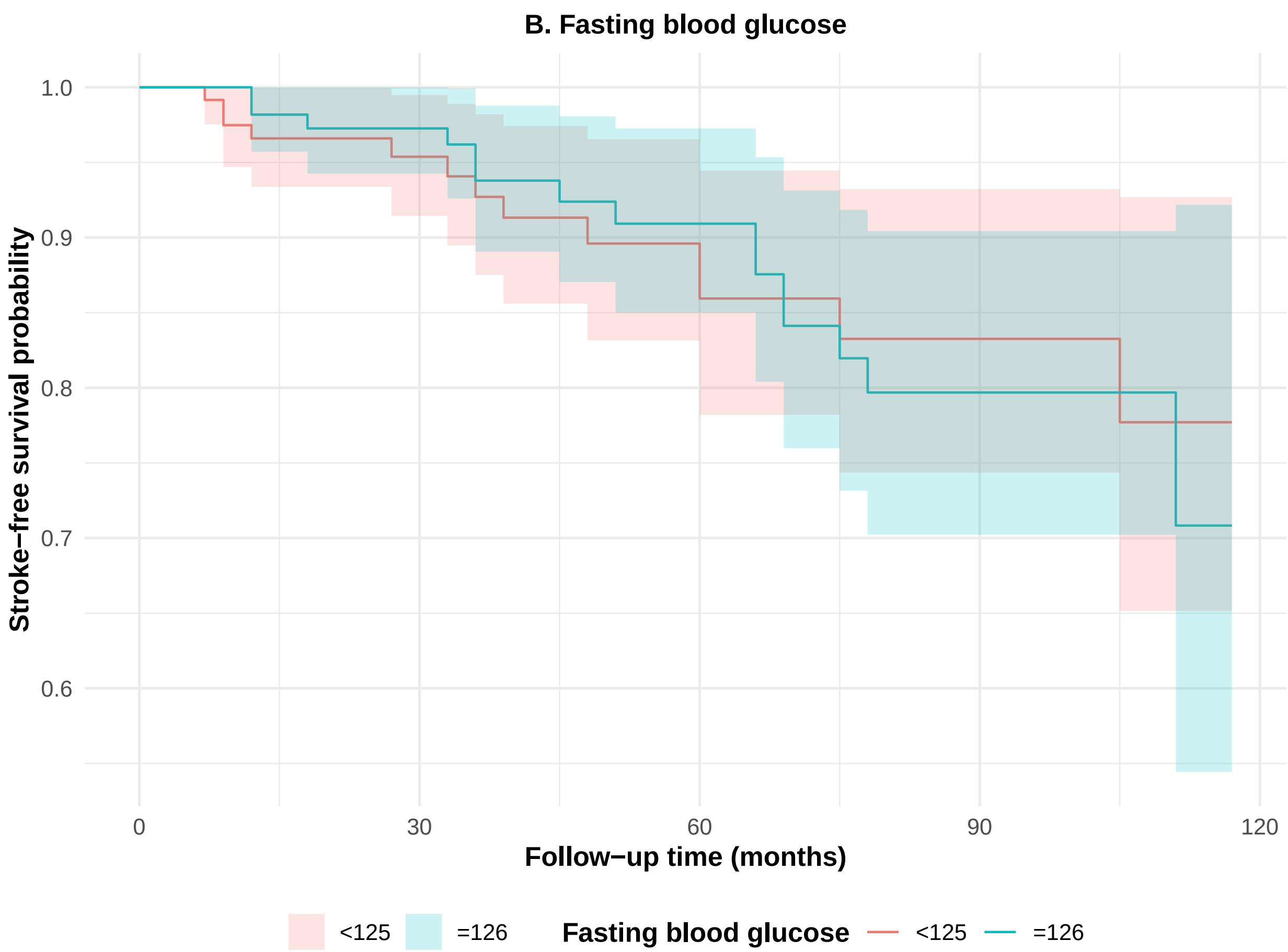
B. Fasting blood glucose
Stroke−free survival probability
1.0
0.9
0.8
0.7
0.6
0
30
60
90
120
Follow−up time (months)
<125
=126
Fasting blood glucose
<125
=126

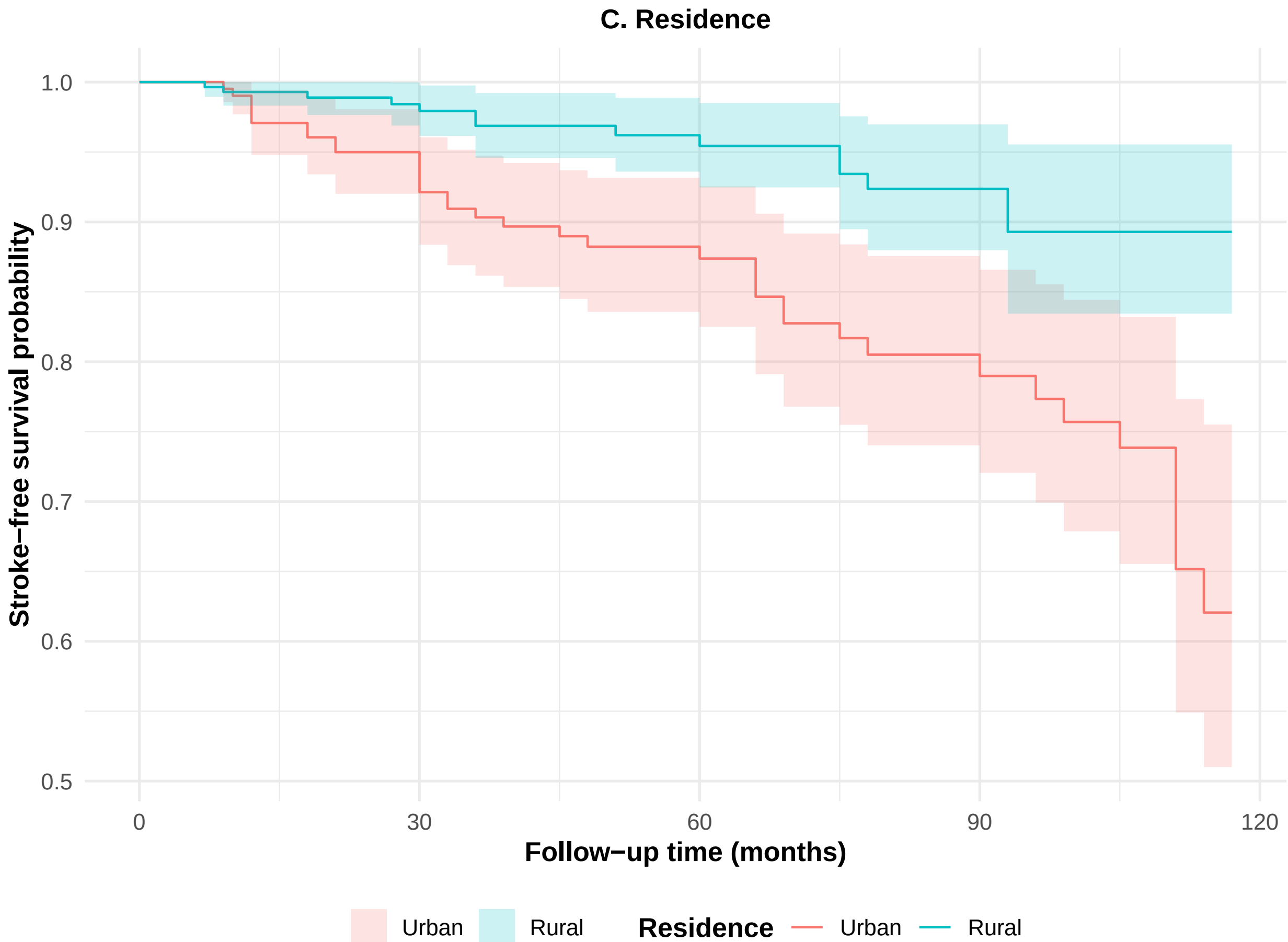
C. Residence
Stroke−free survival probability
1.0
0.9
0.8
0.7
0.6
0.5
0
30
60
90
120
Follow−up time (months)
Urban
Rural
Residence
Urban
Rural

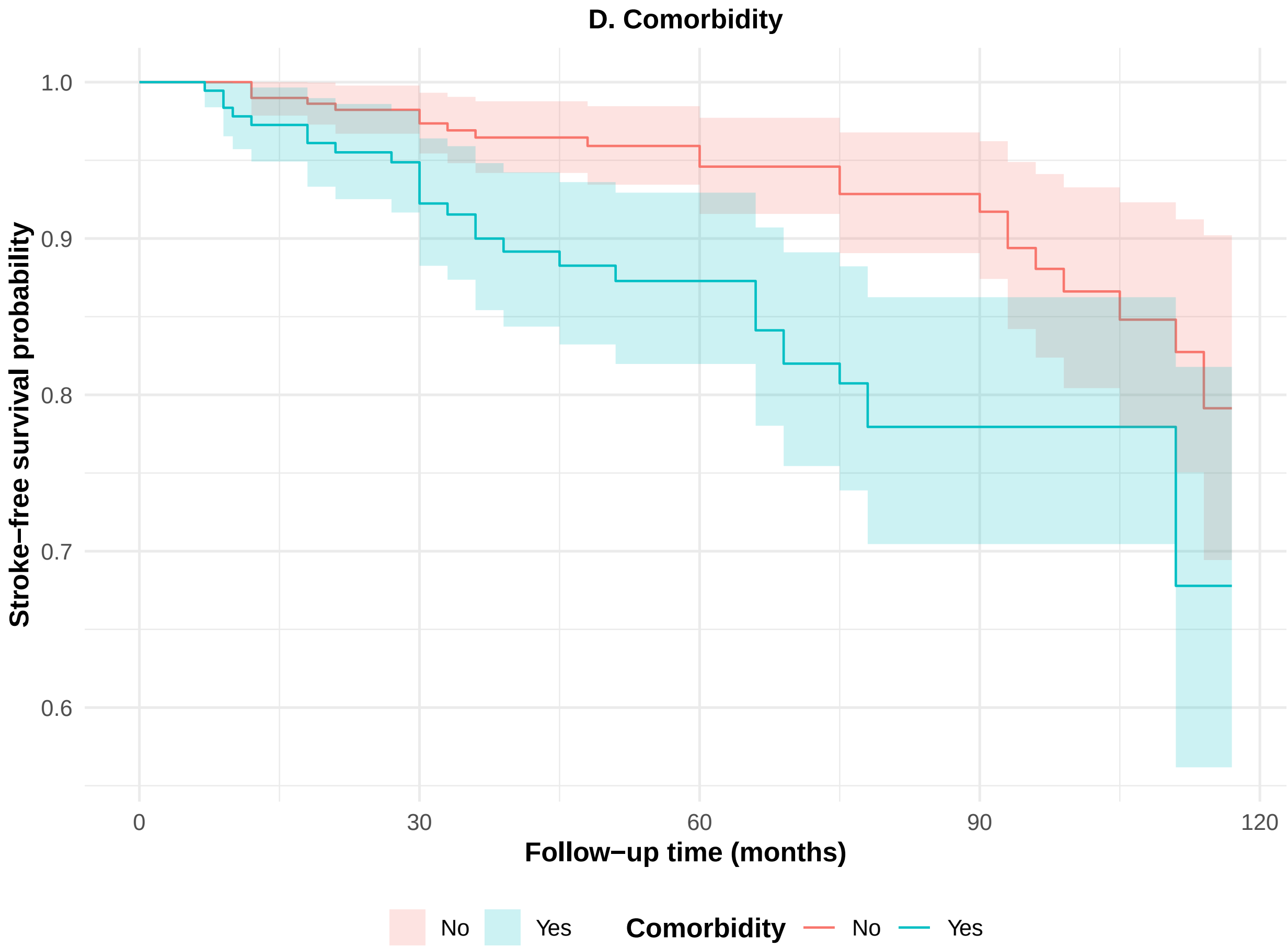

D. Comorbidity
Stroke−free survival probability
1.0
0.9
0.8
0.7
0.6
0
30
60
90
120
Follow−up time (months)
No
Yes
Comorbidity
No
Yes

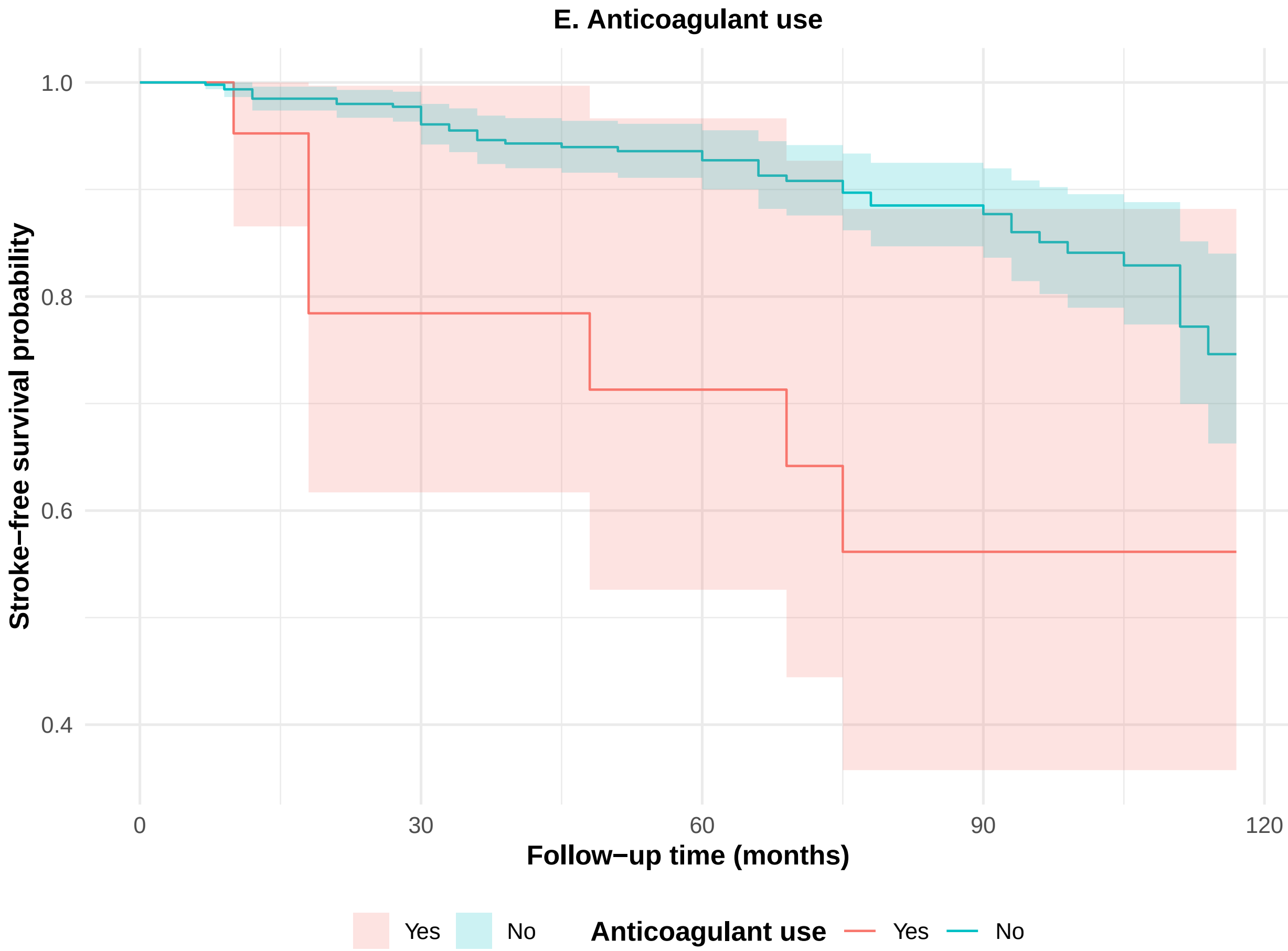
E. Anticoagulant use
Stroke−free survival probability
1.0
0.8
0.6
0.4
0
30
60
90
120
Follow−up time (months)
Yes
No
Anticoagulant use
Yes
No

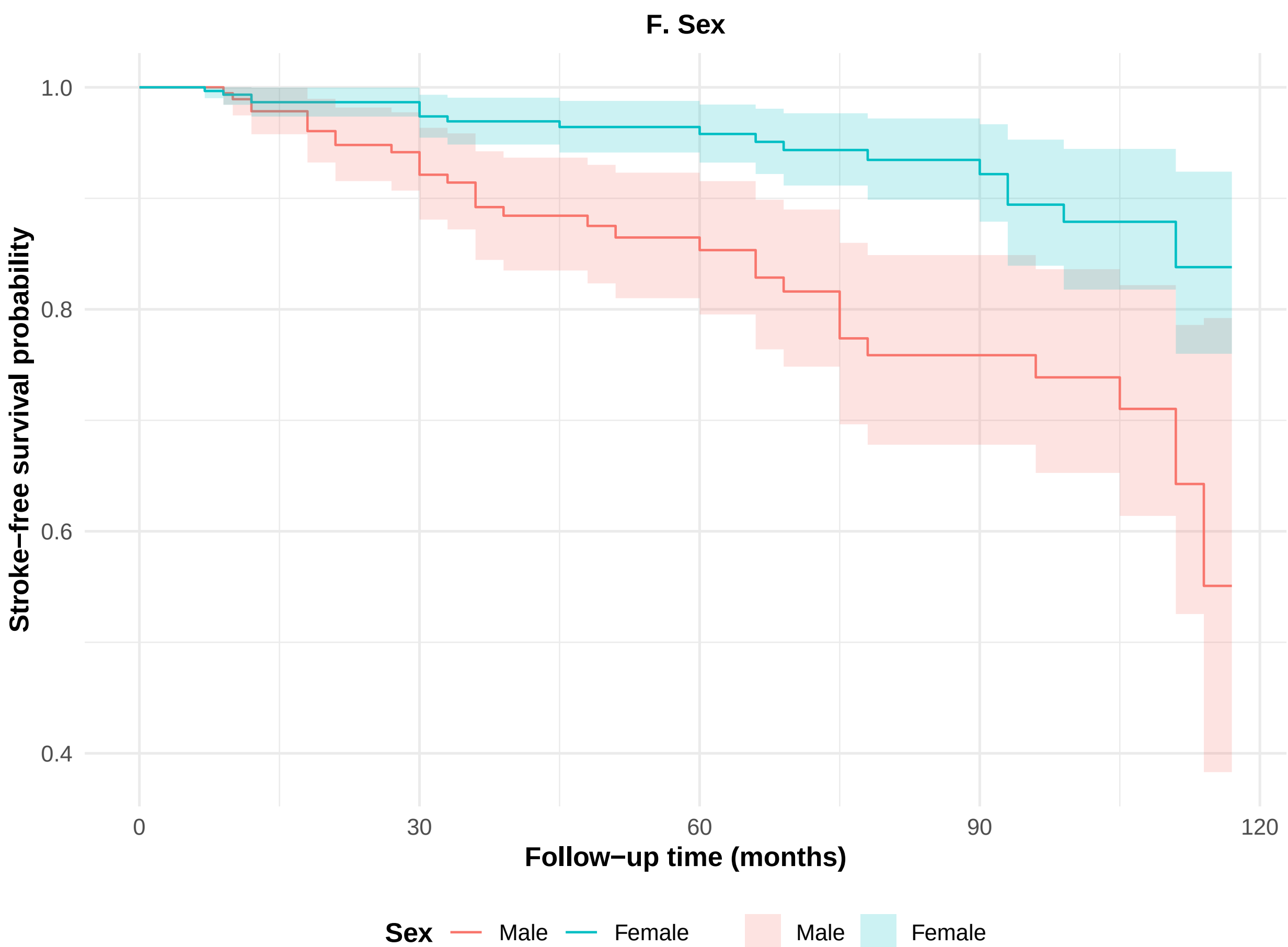
F. Sex
Stroke−free survival probability
1.0
0.8
0.6
0.4
0
30
60
90
120
Follow−up time (months)
Sex
Male
Female
Male
Female

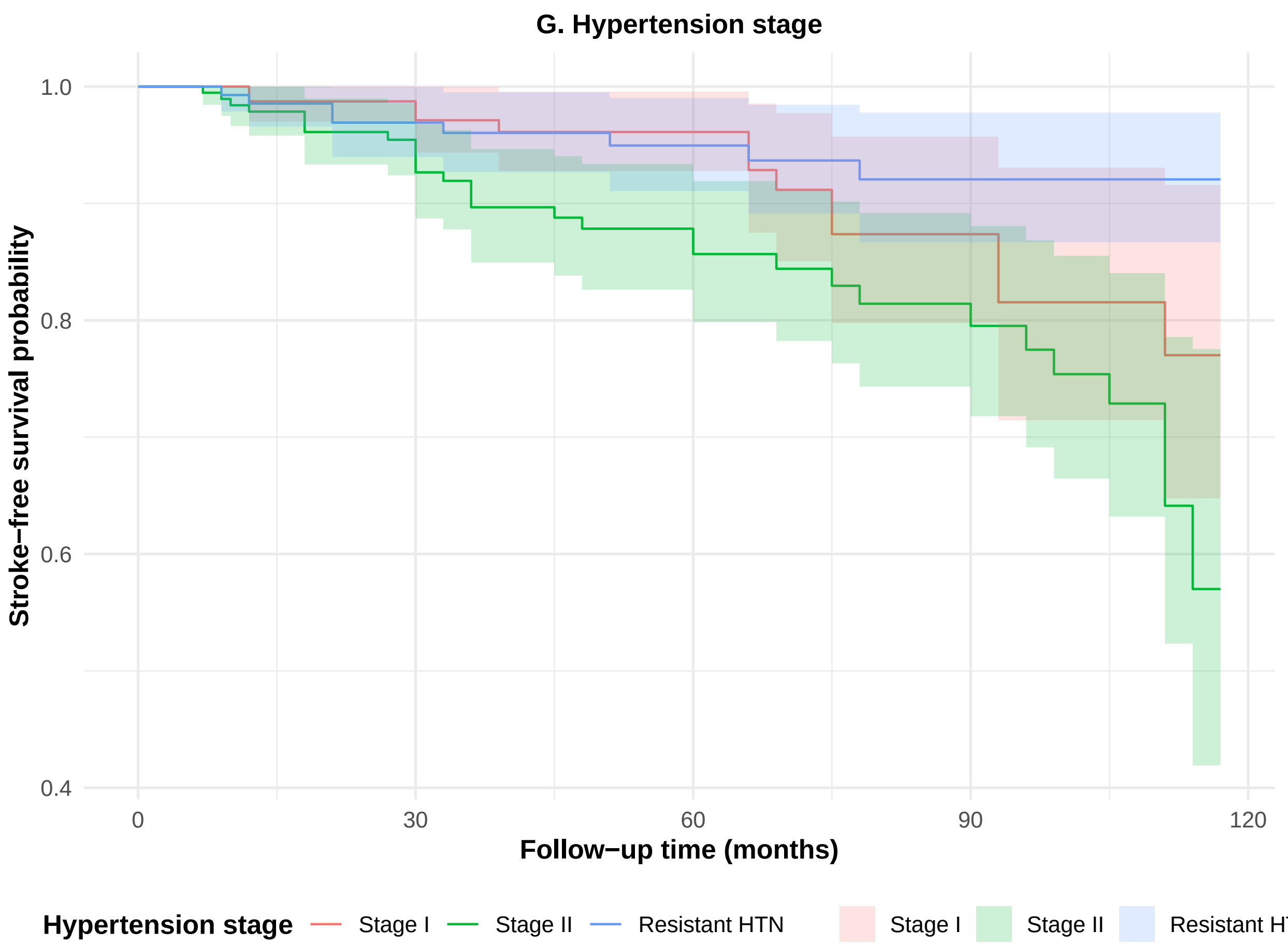

Figure S2. Kaplan–Meier estimates of stroke-free survival according to selected patient characteristics among hypertensive patients in general hospitals of Benishangul-Gumuz, Ethiopia, 2015–2024. (A) age group; (B) fasting blood glucose; (C) residence; (D) comorbidity; (E) anticoagulant use; (F) sex; and (G) hypertension stage. Shaded areas represent 95% confidence intervals.

2 Time to stroke

2.1 Cox proportional hazards regression

A multivariable Cox proportional hazards model was used to evaluate factors associated with time to stroke. Variables with a p-value <0.25 in the bivariable analysis were considered for inclusion in the multivariable model. Associations are presented as hazard ratios (HRs) with corresponding 95% confidence intervals (CIs) and p-values.

Table S1. Multivariable Cox proportional hazards model for time to stroke among hypertensive patients in general hospitals of Benishangul-Gumuz, Ethiopia, 2015–2024.

2.2 Assessment of the proportional hazards assumption

The proportional hazards assumption was assessed using Schoenfeld residuals. Results for individual covariates and the global test are presented in Table S2. A p-value >0.05 was considered consistent with the proportional hazards assumption.

Table 1: Multivariable Cox proportional hazards model for time to stroke

	Variable	HR	CI	P_value
sexFemale	sexFemale	0.40	0.21 - 0.76	0.005
residenceRural	residenceRural	0.29	0.15 - 0.54	<0.001
age_cat 65	age cat greater than or equal to 65	3.64	2.02 - 6.58	<0.001
comorbidYes	comorbidYes	2.32	1.31 - 4.1	0.004
stage_HTNStage II	stage HTNStage II	2.58	1.33 - 5.03	0.005
stage_HTNResistant HTN	stage HTNResistant HTN	0.66	0.27 - 1.62	0.363
Anticoagulant_useNo	Anticoagulant useNo	1.06	0.43 - 2.58	0.904
Tx_Comb Two Regimens	Tx Comb greater than or equal to Two Regimens	0.56	0.28 - 1.09	0.088

Table 2: Schoenfeld residual test for proportional hazards assumption

	Variable	Chi_square	df	P_value
sex	sex	0.05	1	0.828
residence	residence	0.03	1	0.861
age_cat	age_cat	0.27	1	0.600
comorbid	comorbid	1.63	1	0.202
stage_HTN	stage_HTN	3.75	2	0.153
Anticoagulant_use	Anticoagulant_use	3.51	1	0.061
Tx_Comb	Tx_Comb	0.06	1	0.804
GLOBAL	GLOBAL	9.51	8	0.301

Table S2. Schoenfeld residual tests for the proportional hazards assumption.

2.3 Cox–Snell residual diagnostics

Overall model adequacy was assessed using Cox–Snell residuals. The cumulative hazard of the residuals was compared with the expected 45-degree reference line. Close agreement between the two indicates adequate overall model fit.

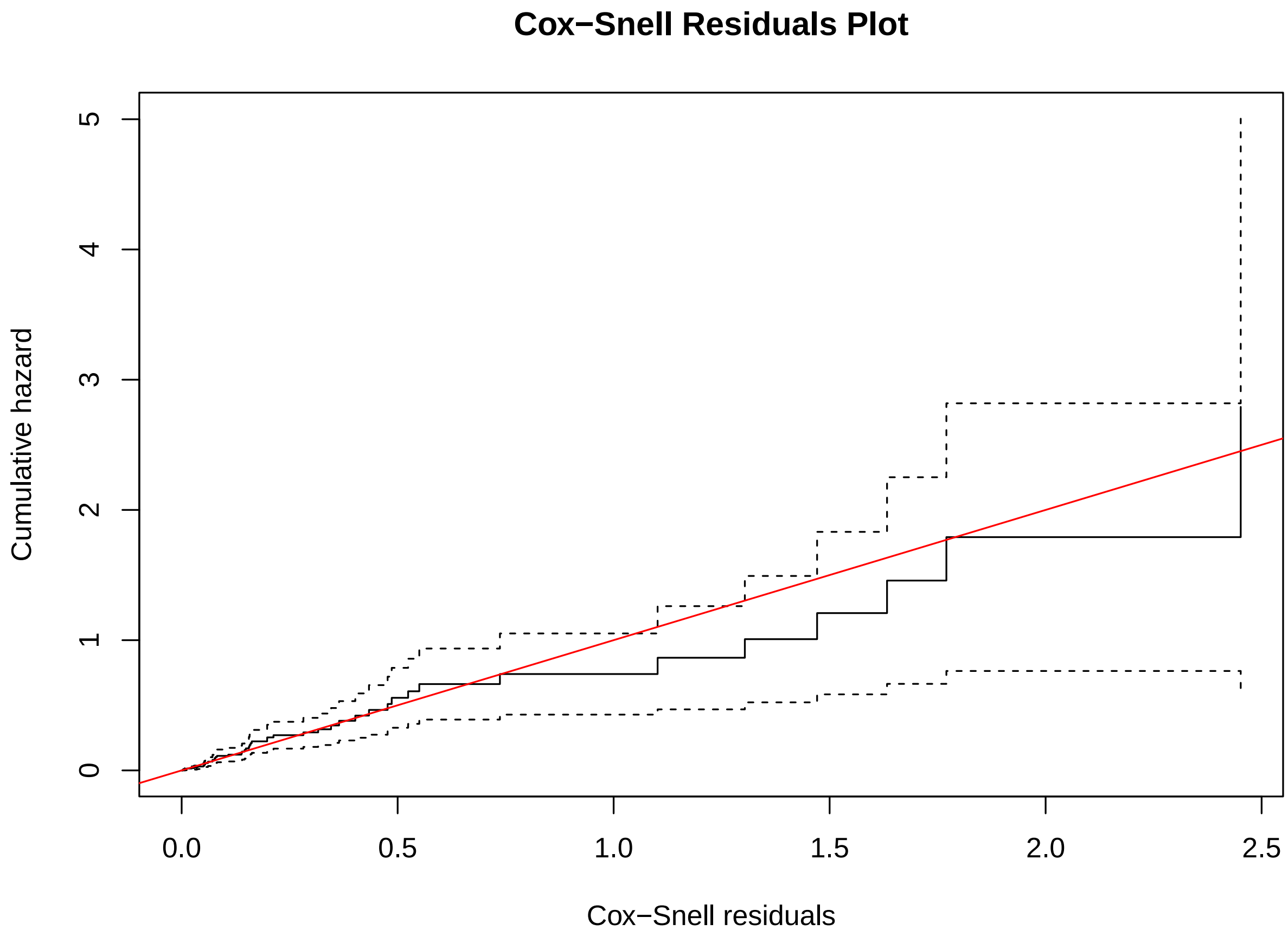

Figure S3. Cox–Snell residual diagnostics for the final Cox proportional hazards model. The estimated cumulative hazard of the Cox–Snell residuals is compared with the expected 45-degree reference line; close agreement indicates adequate overall model fit. # Longitudinal Sub-Models

Longitudinal systolic blood pressure (SBP) and diastolic blood pressure (DBP) trajectories were evaluated using linear mixed-effects models with three alternative random-effects specifications: random intercept only, random intercept and slope, and a flexible random spline specification. Model adequacy was assessed using the Akaike information criterion (AIC), Bayesian information criterion (BIC), and log-likelihood. Distributional assumptions and potential outliers were also assessed before model fitting.

For SBP, the random spline model provided the best relative fit among the models considered (AIC = 21,489.60; BIC = 21,560.68), with a substantial improvement over the random-intercept model (ΔAIC = 204.21) and a similar fit to the random intercept-and-slope model.

For DBP, the random spline model provided a substantially better fit than both the random-intercept model (ΔAIC = 520.24) and the random intercept-and-slope model (ΔAIC = 301.92), based on AIC. Accordingly, the random spline specification was selected for the longitudinal SBP and DBP sub-models used in the subsequent Bayesian joint modeling analysis. ## Model Comparison for SBP

Table S3. Comparison of linear mixed-effects models for longitudinal systolic blood pressure

3 Bayesian Multivariate Joint Modeling

Following selection of the longitudinal and survival sub-models, a Bayesian multivariate joint modeling framework was fitted using Markov chain Monte Carlo (MCMC) methods to jointly model longitudinal SBP

Table 3: Comparison of alternative longitudinal models for systolic blood pressure

Model	AIC	BIC
Random intercept	21689.81	21746.67
Random intercept + slope	21489.60	21560.68
Random spline	21489.60	21560.68

Table 4: Comparison of alternative longitudinal models for diastolic blood pressure

Model	AIC	BIC
Random intercept	16194.95	16251.81
Random intercept + slope	15976.63	16047.70
Random spline	15674.71	15795.53

and DBP trajectories and time to stroke. The joint model accounted for the correlation between the repeated blood pressure measurements and the time-to-event outcome.

Four alternative association structures were evaluated: current value, current slope, current value plus slope, and cumulative average. Model fit was compared using the conditional Deviance Information Criterion (DIC), conditional Widely Applicable Information Criterion (WAIC), and conditional Log Pseudo-Marginal Likelihood (LPML). Lower DIC and WAIC values and higher LPML values indicate better relative model fit. The model with the current-value association structure provided the best overall fit and was therefore selected as the primary joint model for inference.

Table S5. Comparison of alternative association structures for the Bayesian multivariate joint model

```
##                Length Class     Mode
## mcmc           14     -none-    list
## acc_rates      11     -none-    list
## running_time    5     proc_time numeric
## statistics     10     -none-    list
## fit_stats       2     -none-    list
## Wlong_bar       2     -none-    list
## Wlong_sds       2     -none-    list
## Wlong_std       2     -none-    list
## W_bar           8     -none-    numeric
## W_sds           8     -none-    numeric
## W_std           8     -none-    numeric
## model_data     58     -none-    list
## model_info     22     -none-    list
## initial_values 12     -none-    list
```

Table 5: Comparison of alternative association structures for Bayesian multivariate joint model

Association_structure	DIC	WAIC	LPML
Current value	40390.30	40186.19	-21237.69
Current slope	40528.05	40256.31	-21253.64
Current value + slope	40613.22	40355.54	-25794.90
Cumulative average	40871.92	40479.85	-21346.17

```
## control        15     -none-    list
## priors         49     -none-    list
## call            9     -none-    call
```

3.1 Bayesian Model Convergence and Diagnostics

Convergence of the selected Bayesian multivariate joint model was assessed using trace plots and the potential scale reduction factor (R-hat). The trace plots showed stable mixing of the Markov chains without apparent trends or prolonged periods of poor mixing. R-hat values were close to 1.00, with values 1.01 for the assessed parameters, indicating satisfactory convergence of the corresponding MCMC chains. These diagnostics supported the adequacy of the posterior sampling for the selected joint model.

Figure S4. Trace plots for selected parameters of the Bayesian multivariate joint model

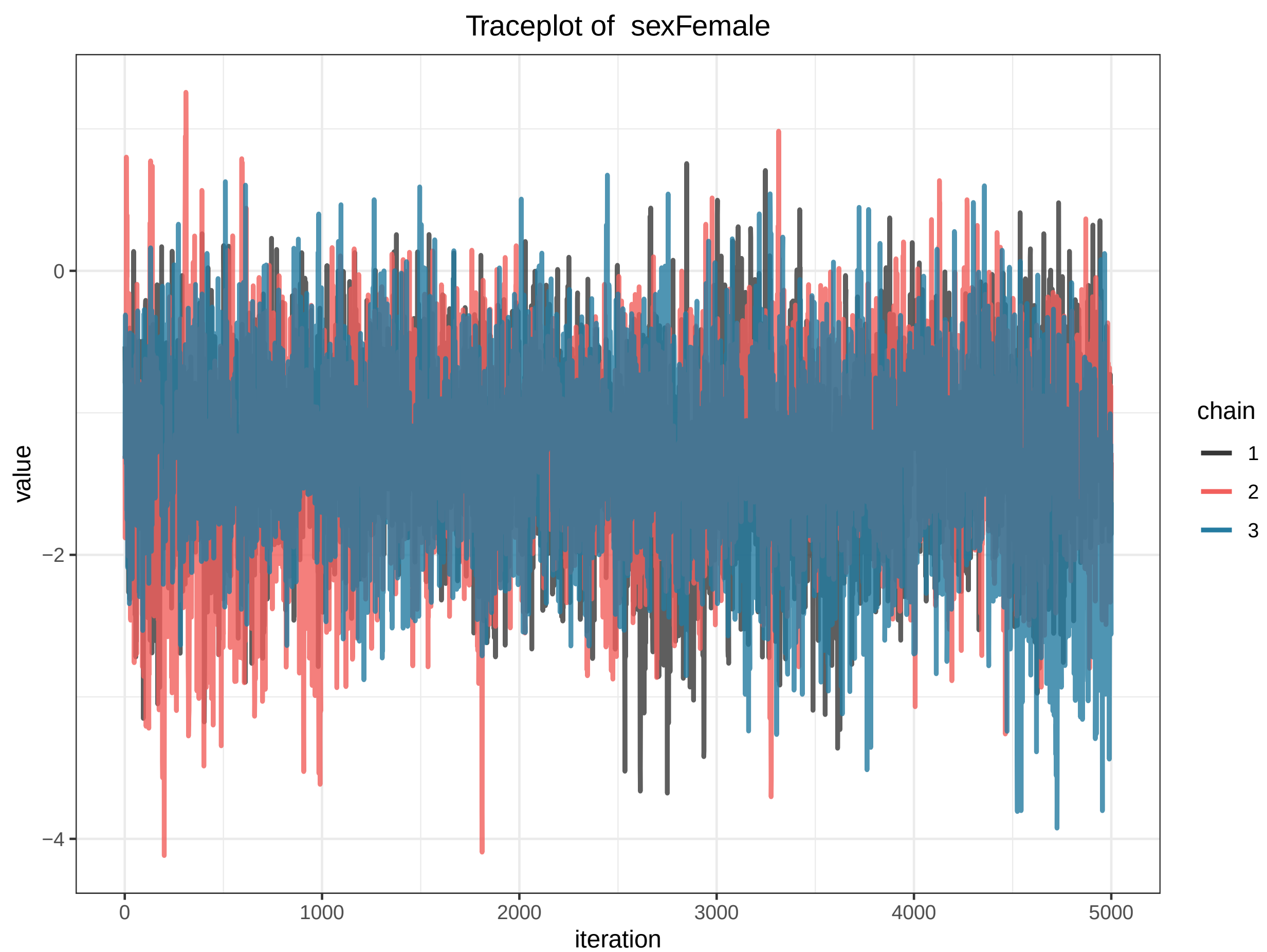

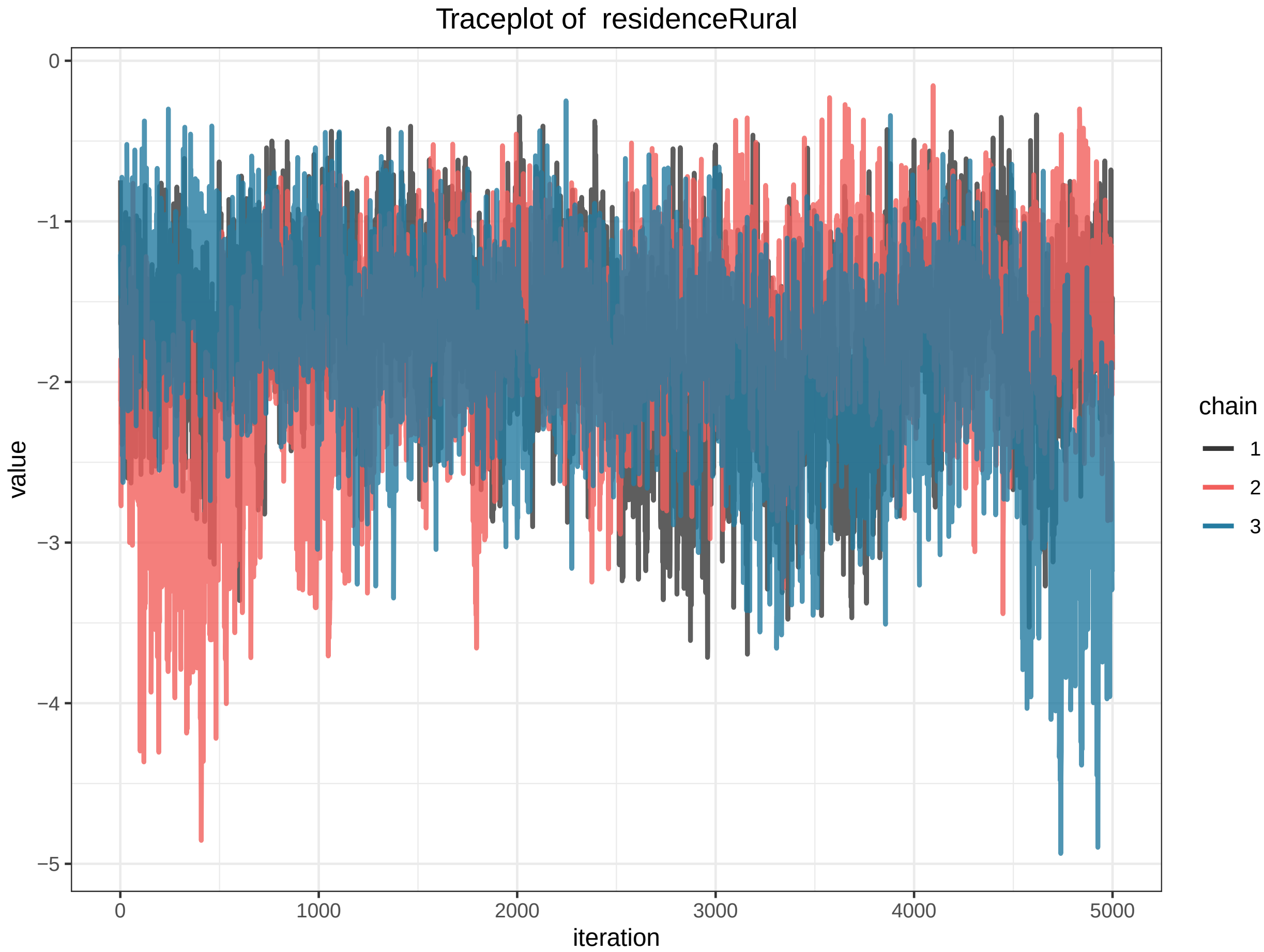

Traceplot of residenceRural
value
iteration
chain
1
2
3
0
−1
−2
−3
−4
−5
0
1000
2000
3000
4000
5000

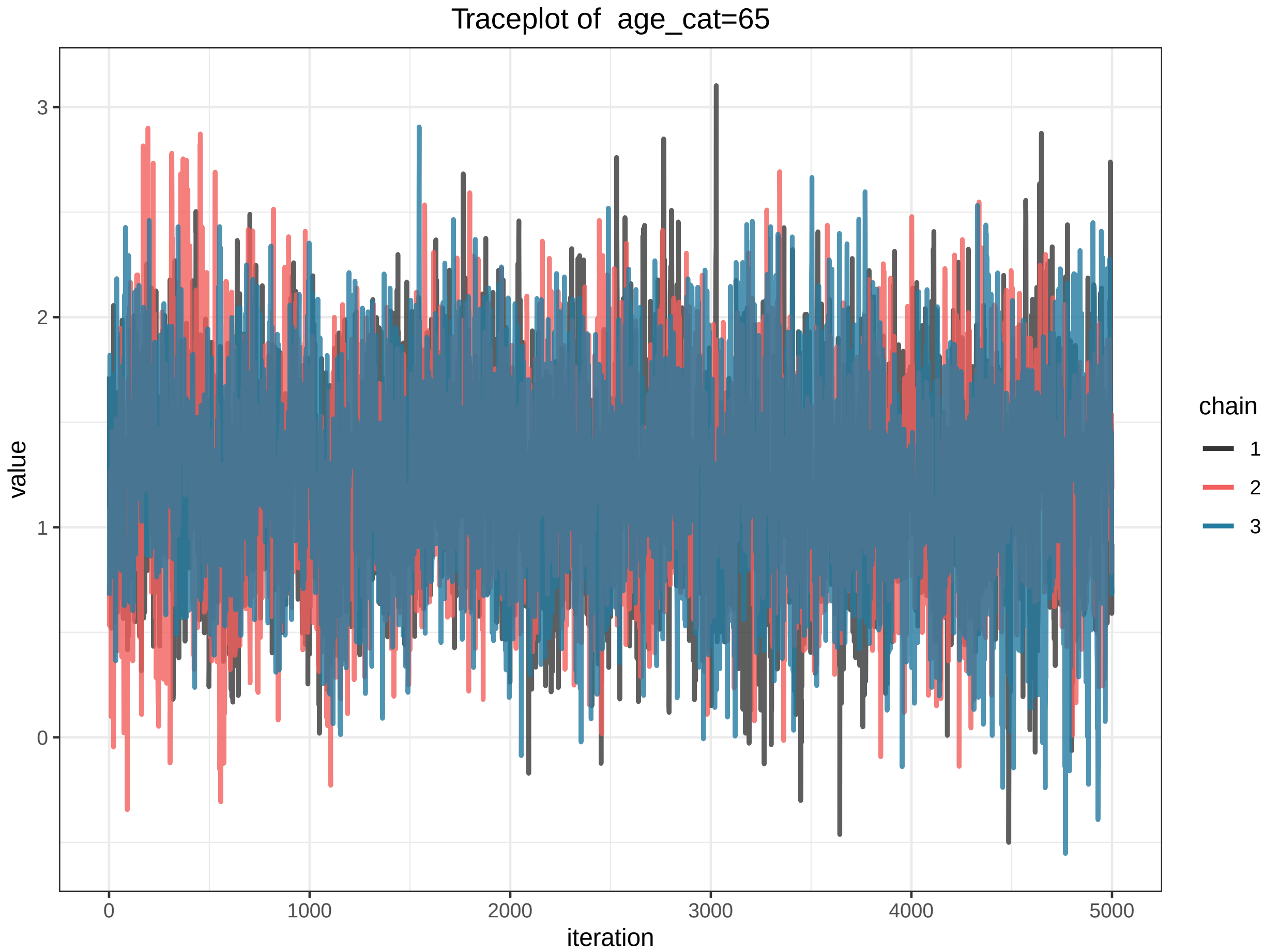

Traceplot of age_cat=65
value
iteration
chain
1
2
3
0
1000
2000
3000
4000
5000

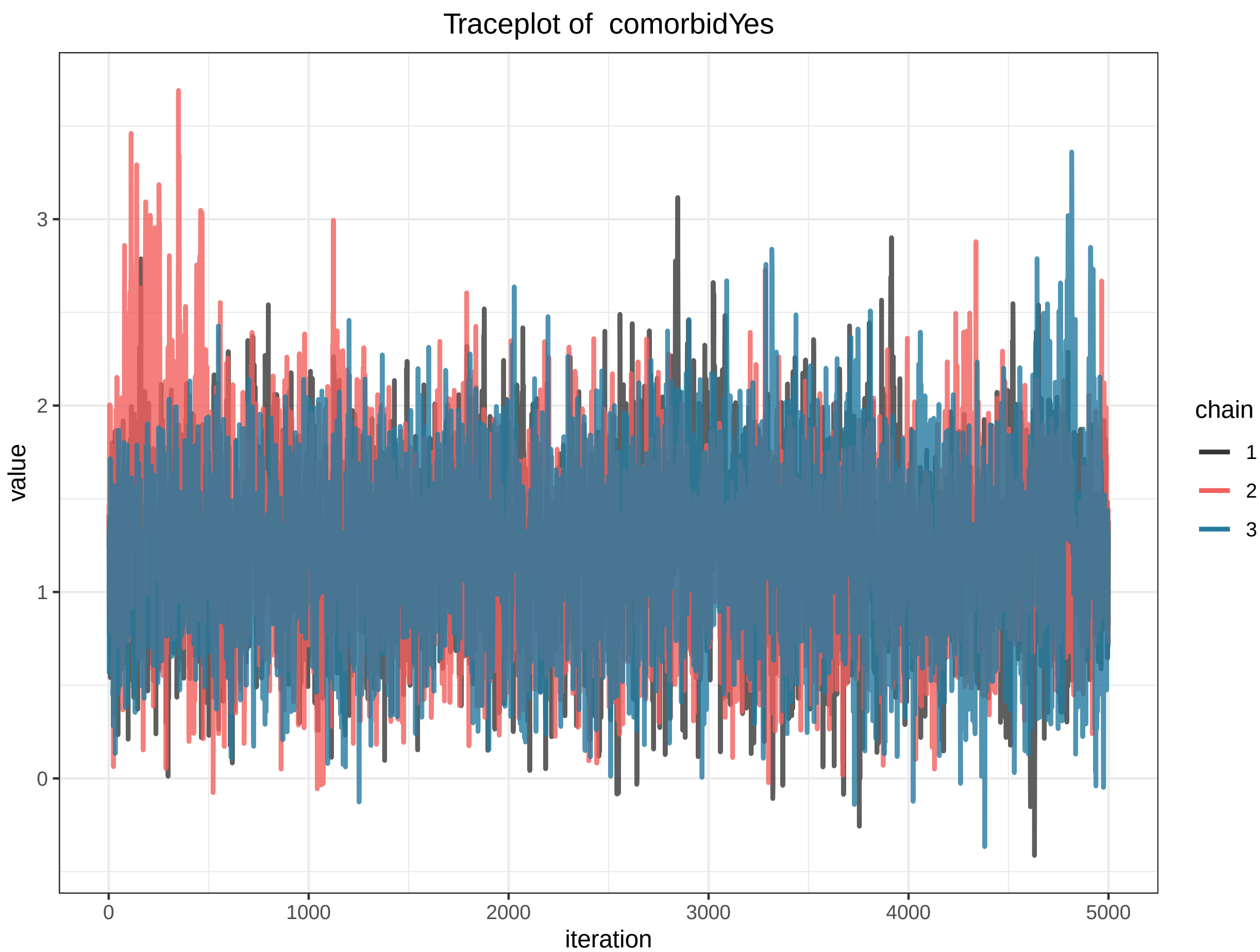

Traceplot of comorbidYes
value
iteration
chain
1
2
3
0
1
2
3
0
1000
2000
3000
4000
5000

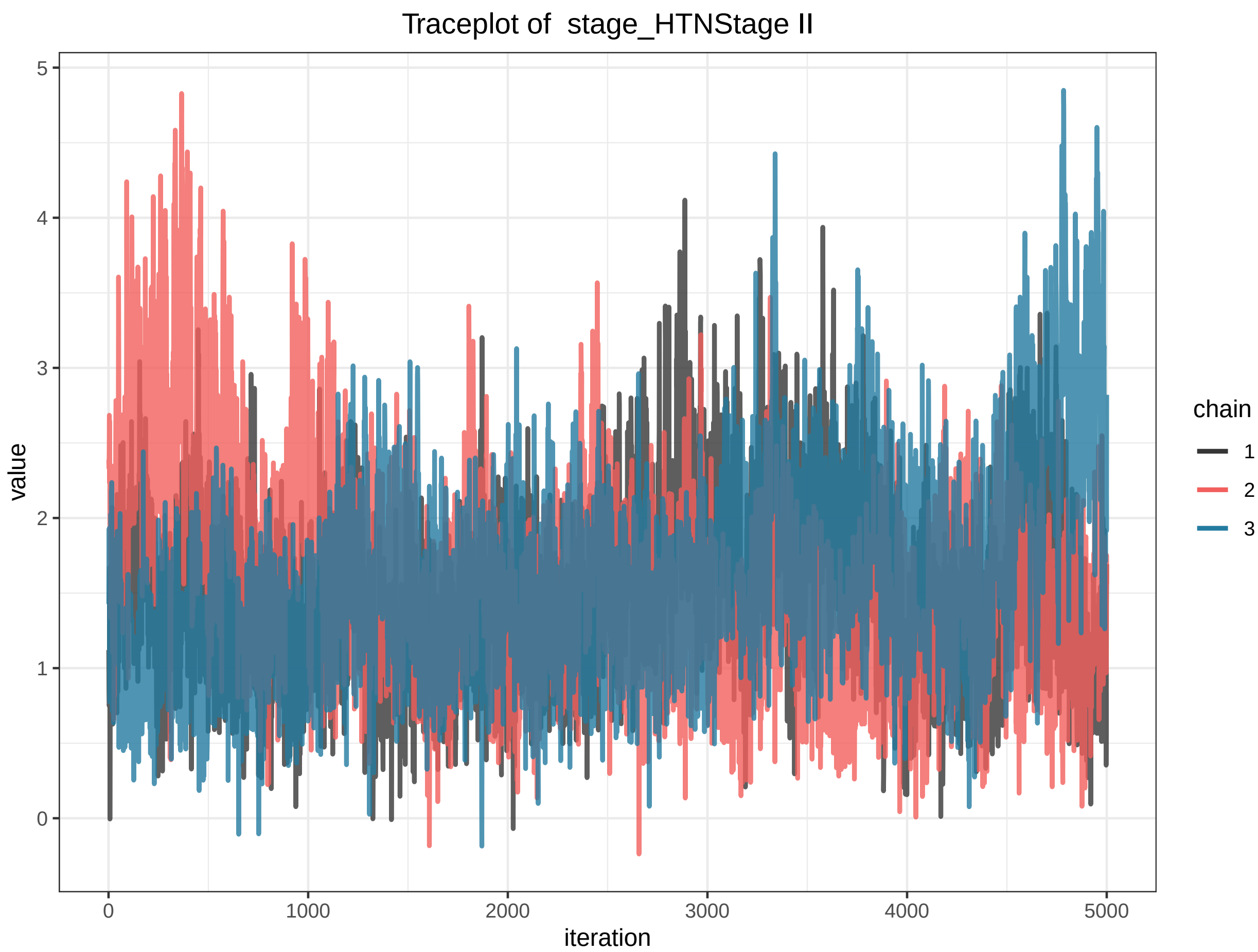
Traceplot of stage_HTNStage II
value
iteration
chain
1
2
3
0
1000
2000
3000
4000
5000
0
1
2
3
4
5

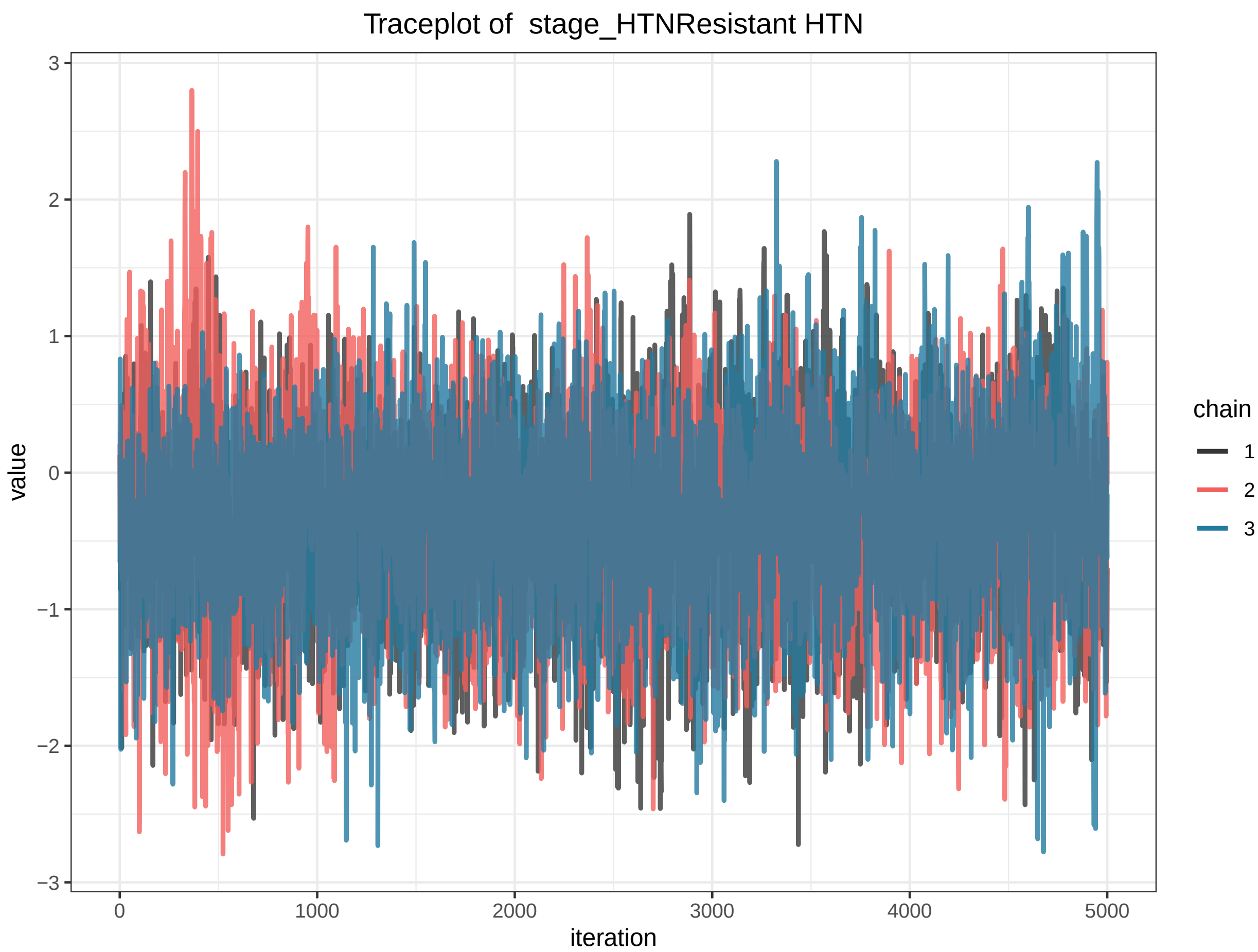
Traceplot of stage_HTNResistant HTN
value
iteration
chain
1
2
3
3
2
1
0
−1
−2
−3
0
1000
2000
3000
4000
5000

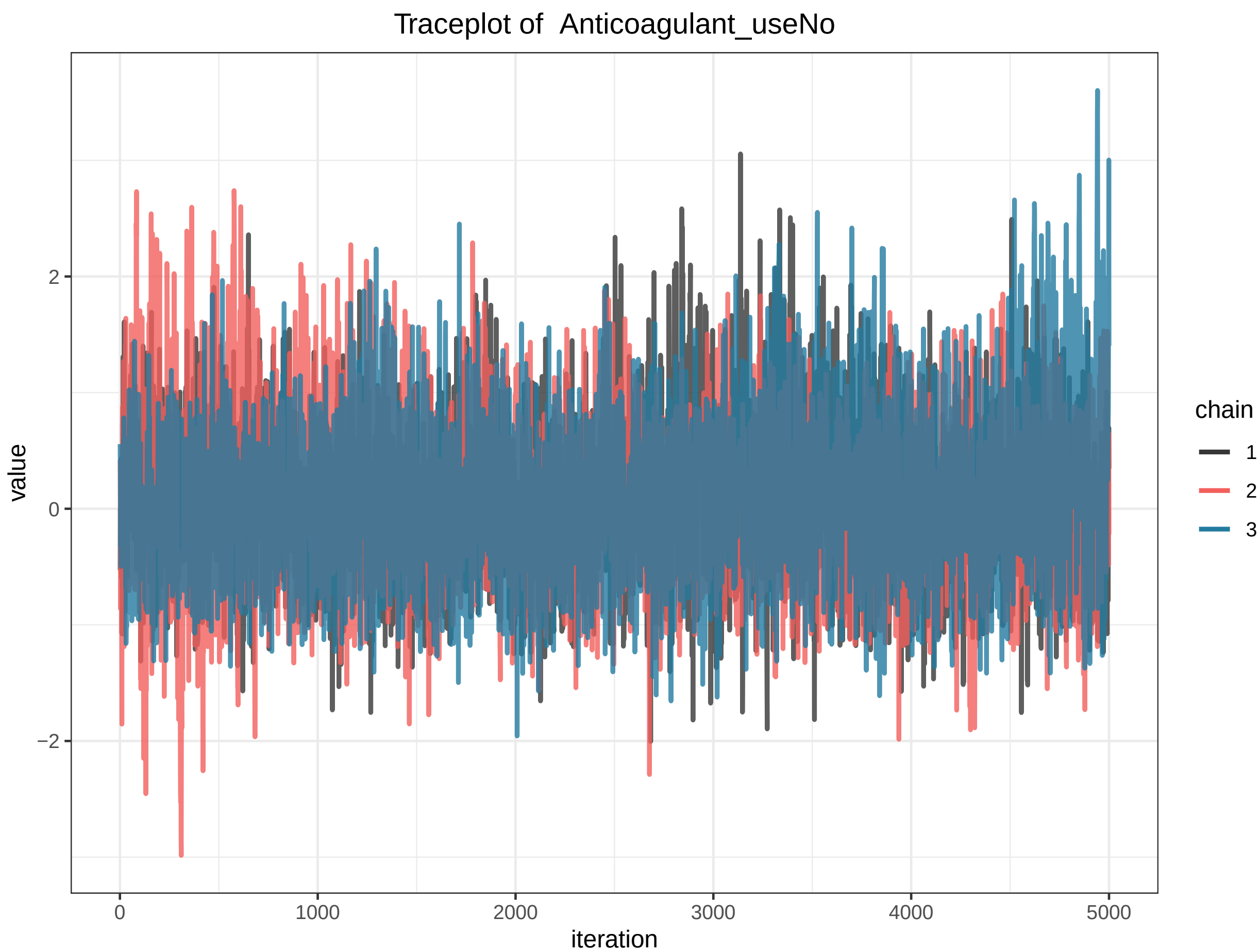

Traceplot of Anticoagulant_useNo
value
2
0
−2
0
1000
2000
3000
4000
5000
iteration
chain
1
2
3

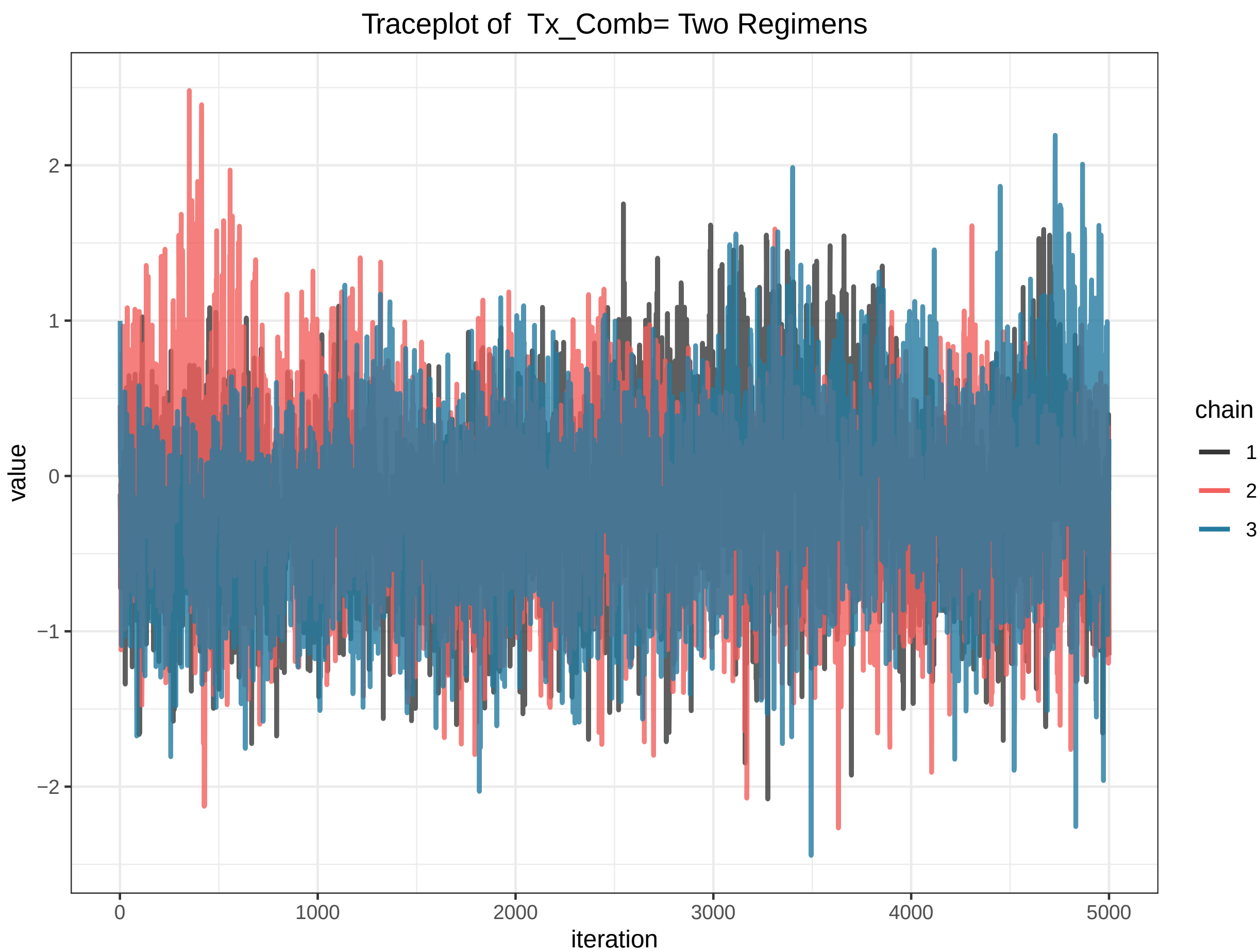


```
## $sqrt_SBP
## Potential scale reduction factors:
##
##                        Point est. Upper C.I.
## (Intercept)                  1.03       1.11
## visit                        1.05       1.16
## sexFemale                    1.00       1.01
## age_cat 65                  1.00       1.01
## comorbidYes                  1.01       1.03
## Tx_Comb  Two Regimens       1.01       1.02
##
## Multivariate psrf
##
## 1.05
##
## $sqrt_DBP
## Potential scale reduction factors:
##
##                        Point est. Upper C.I.
## (Intercept)                  1.02       1.06
## visit                        1.02       1.05
## sexFemale                    1.00       1.00
## age_cat 65                  1.00       1.00
## comorbidYes                  1.01       1.02
```

```
## Tx_Comb  Two Regimens       1.01       1.02
##
## Multivariate psrf
##
## 1.02
```

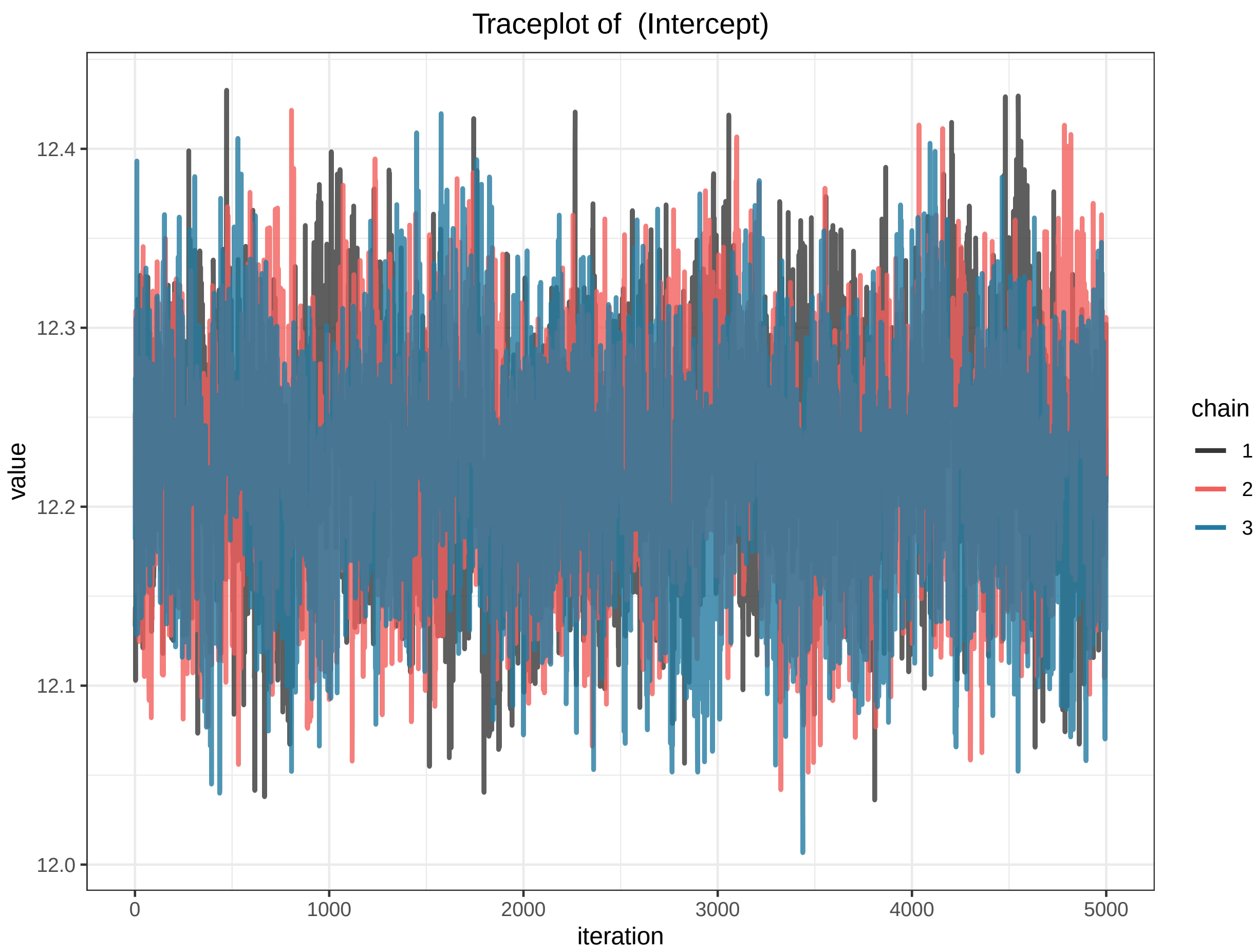

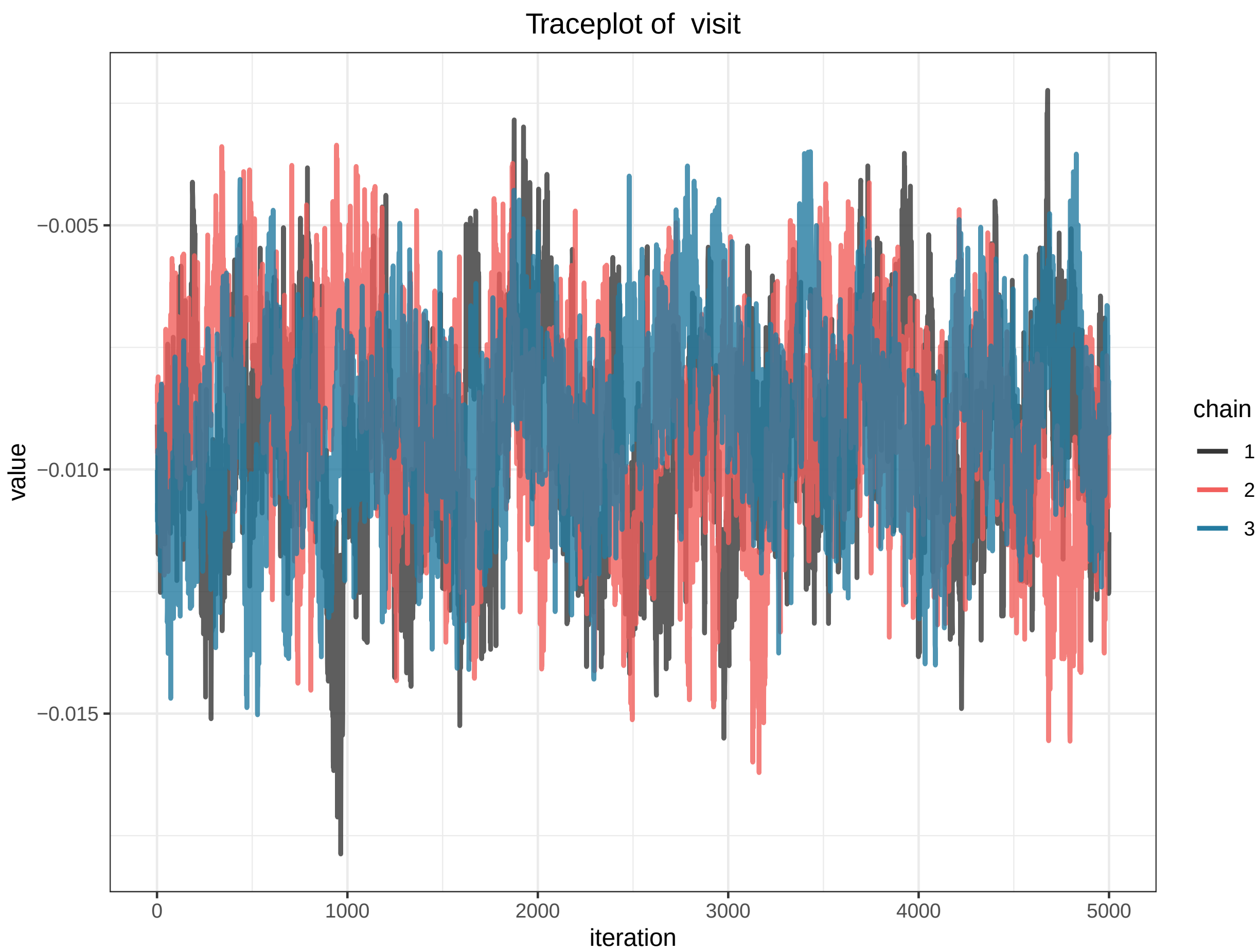

Traceplot of visit
value
−0.005
−0.010
−0.015
0
1000
2000
3000
4000
5000
iteration
chain
1
2
3

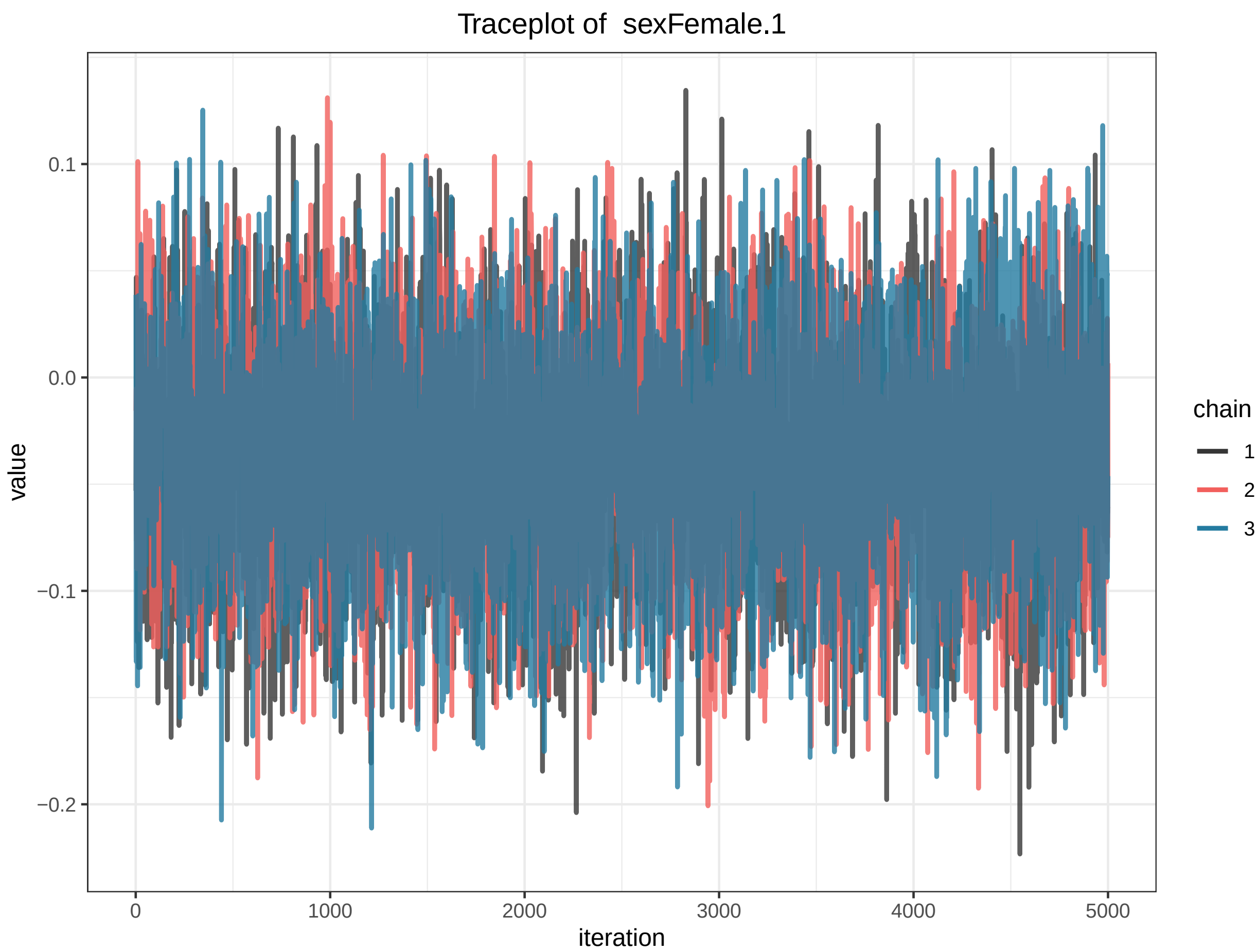
Traceplot of sexFemale.1
value
iteration
0.1
0.0
−0.1
−0.2
0
1000
2000
3000
4000
5000
chain
1
2
3

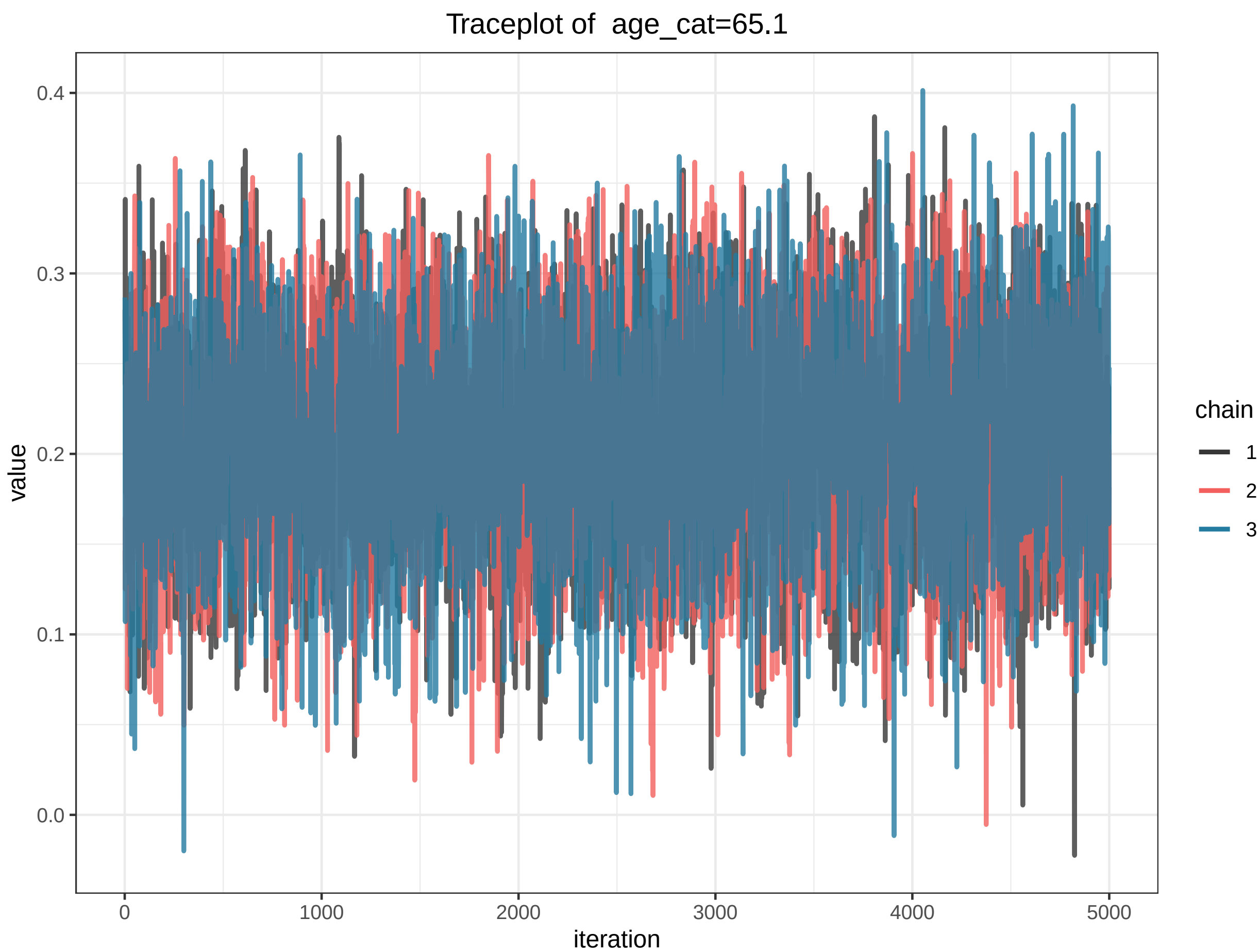
Traceplot of age_cat=65.1
value
0.4
0.3
0.2
0.1
0.0
0
1000
2000
3000
4000
5000
iteration
chain
1
2
3

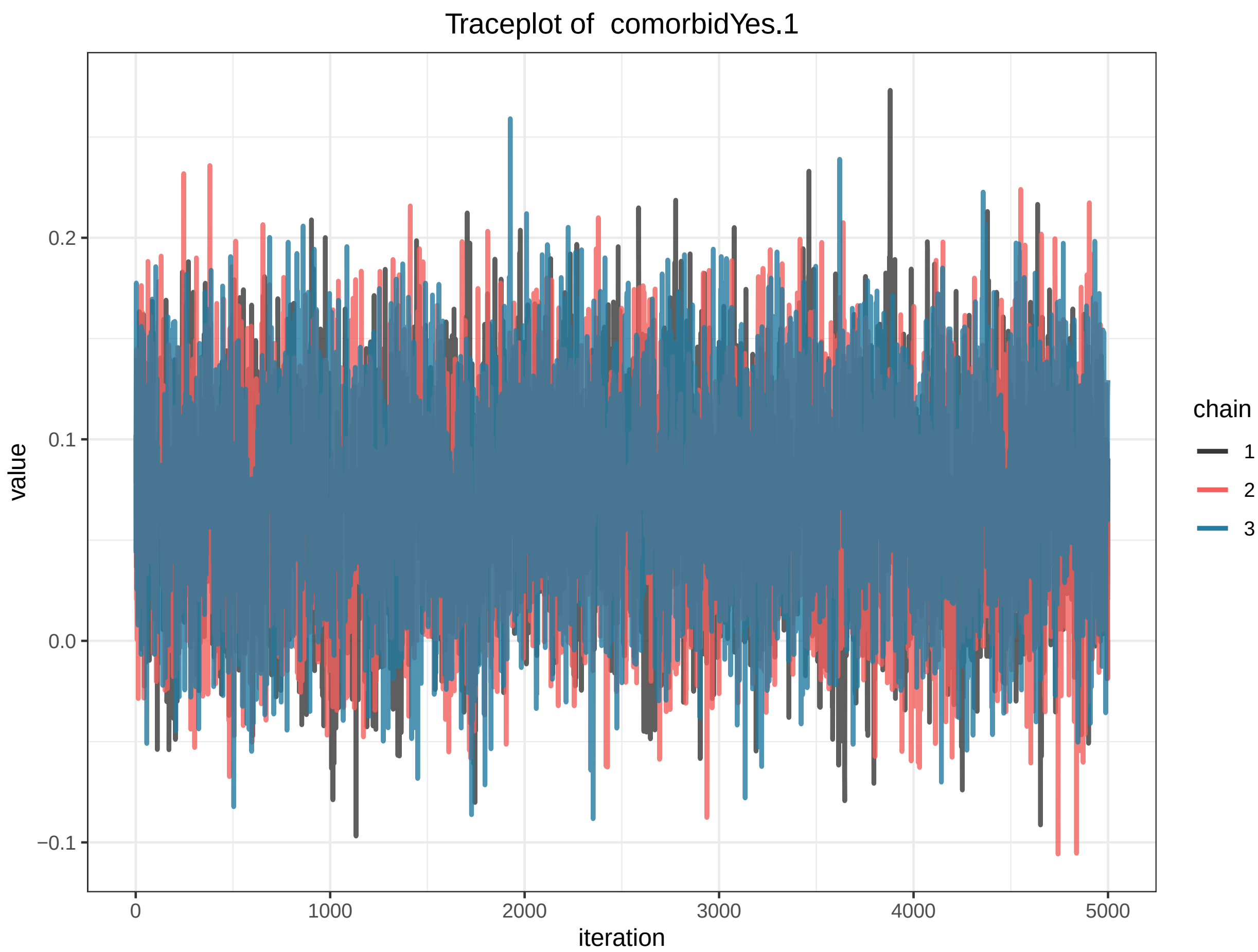
Traceplot of comorbidYes.1
value
0.2
0.1
0.0
−0.1
0
1000
2000
3000
4000
5000
iteration
chain
1
2
3

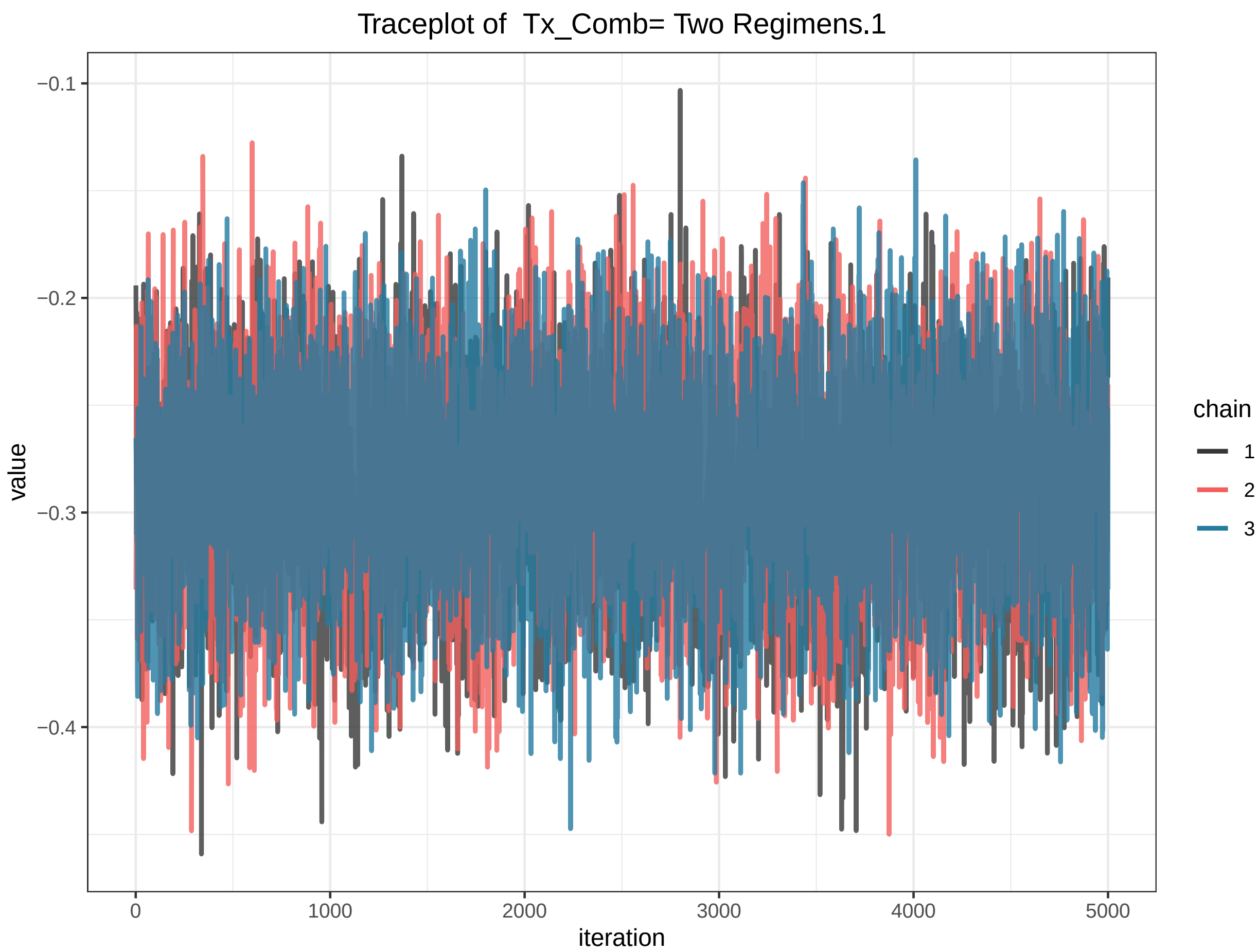

Traceplot of Tx_Comb= Two Regimens.1
value
−0.1
−0.2
−0.3
−0.4
0
1000
2000
3000
4000
5000
iteration
chain
1
2
3

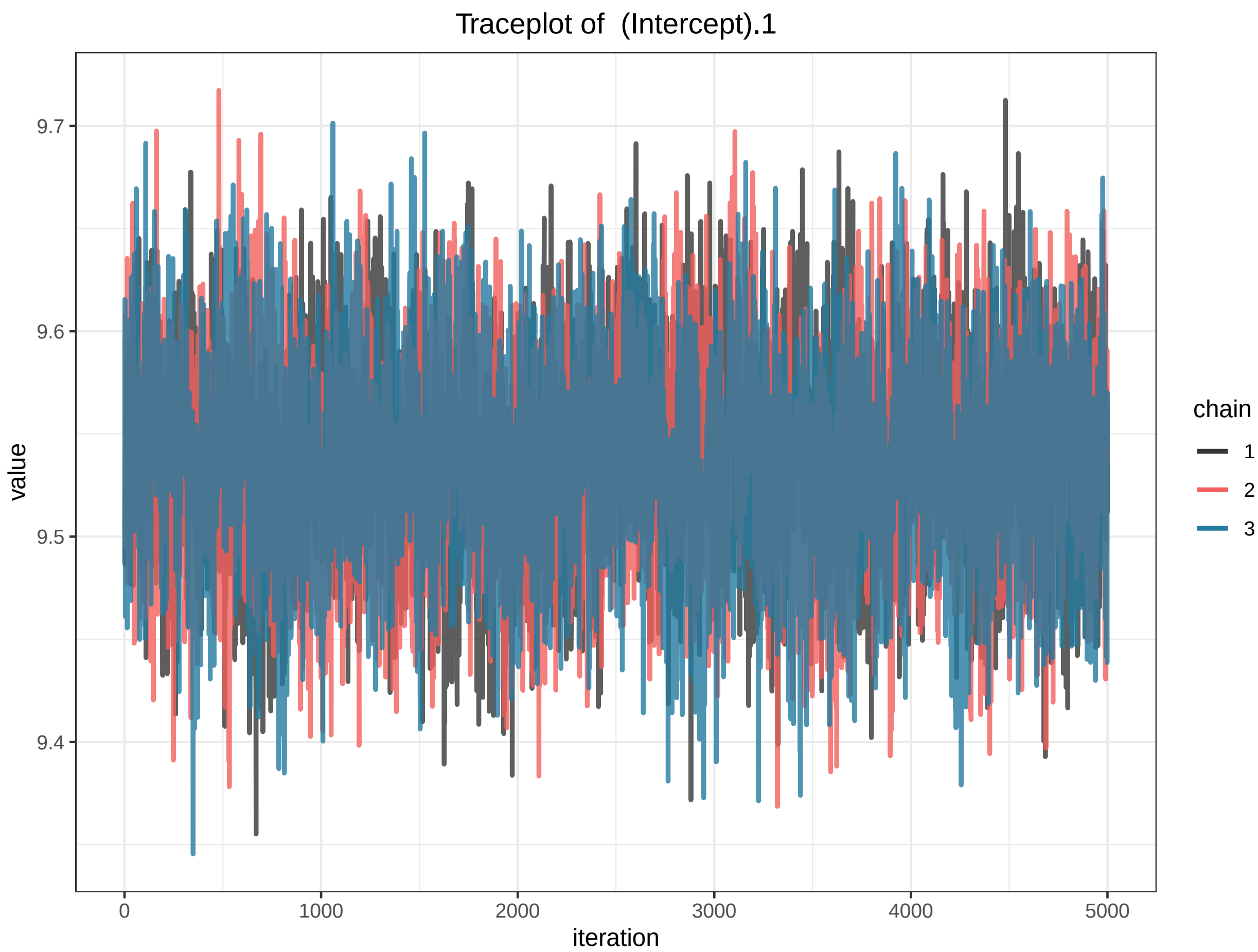
Traceplot of (Intercept).1
value
9.7
9.6
9.5
9.4
0
1000
2000
3000
4000
5000
iteration
chain
1
2
3

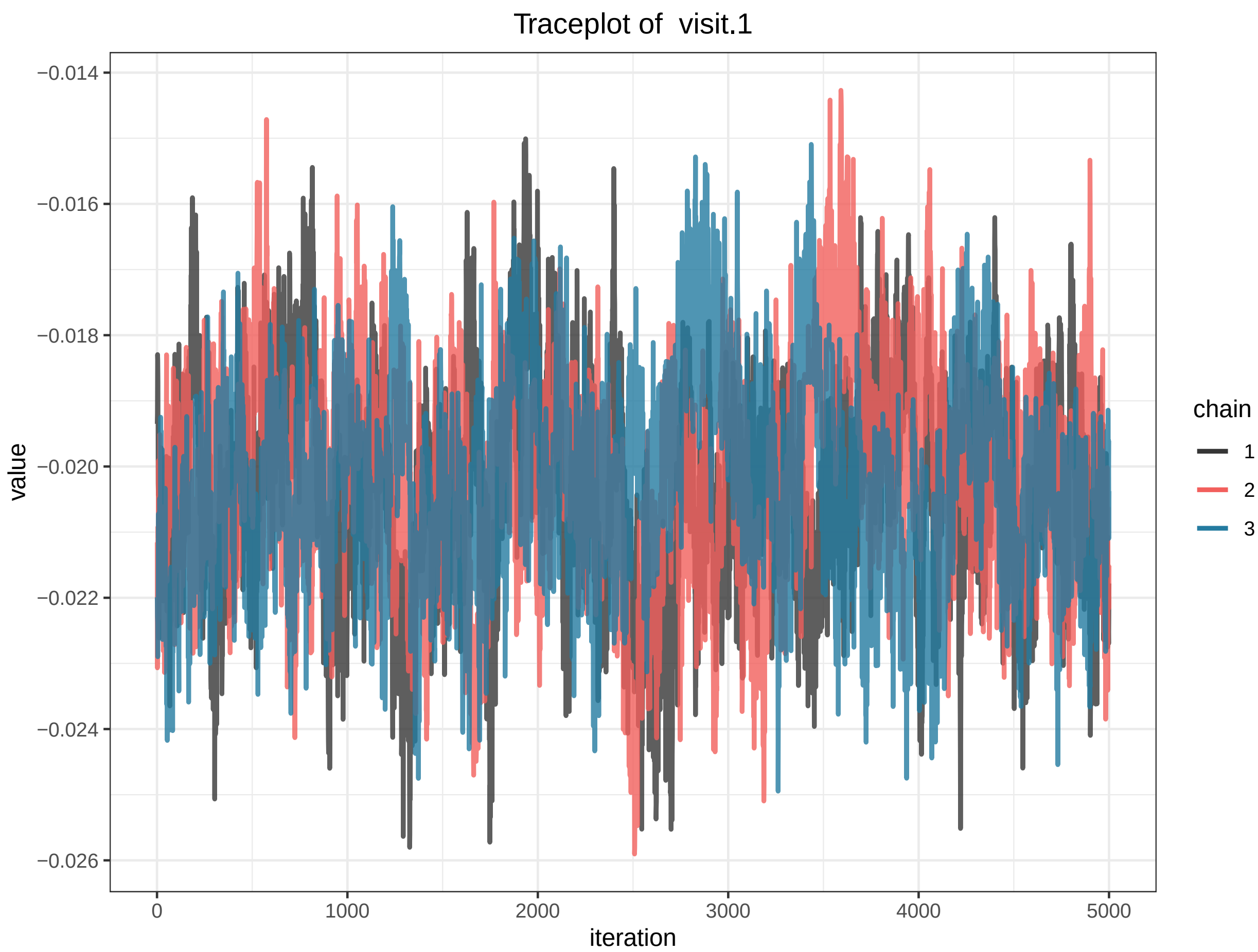

Traceplot of visit.1
−0.014
−0.016
−0.018
−0.020
−0.022
−0.024
−0.026
value
0
1000
2000
3000
4000
5000
iteration
chain
1
2
3

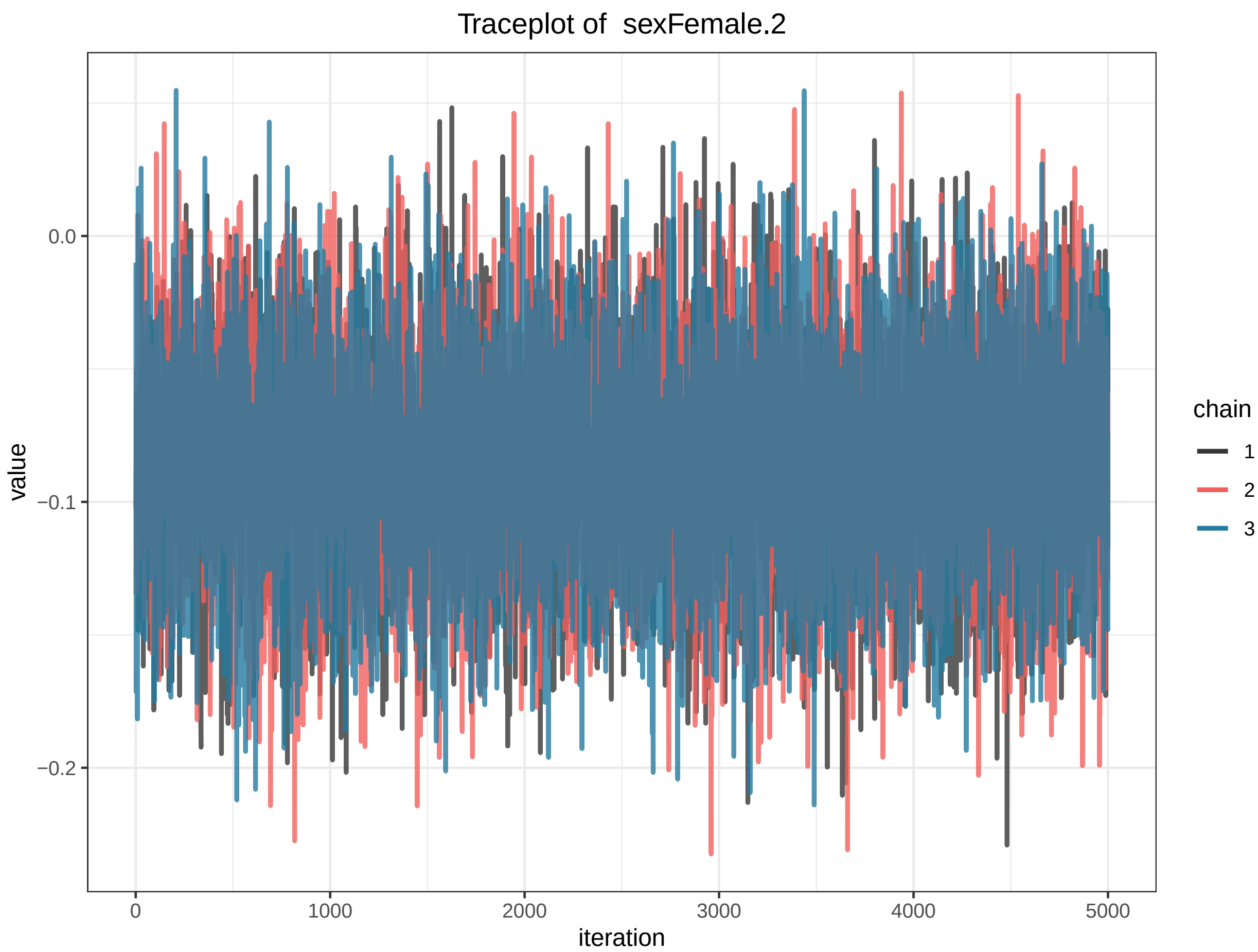
Traceplot of sexFemale.2
value
0.0
−0.1
−0.2
0
1000
2000
3000
4000
5000
iteration
chain
1
2
3

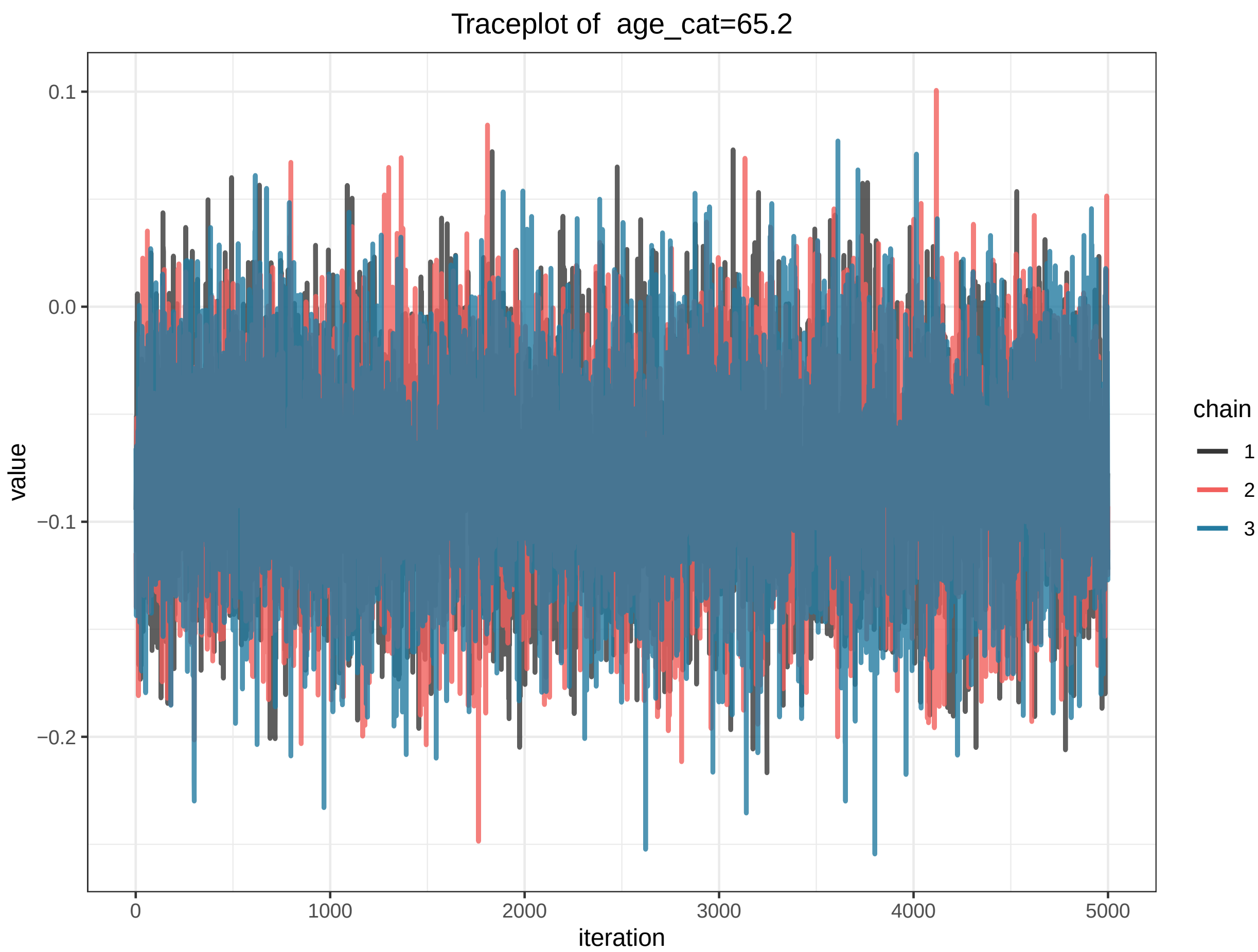
Traceplot of age_cat=65.2
value
0.1
0.0
−0.1
−0.2
0
1000
2000
3000
4000
5000
iteration
chain
1
2
3

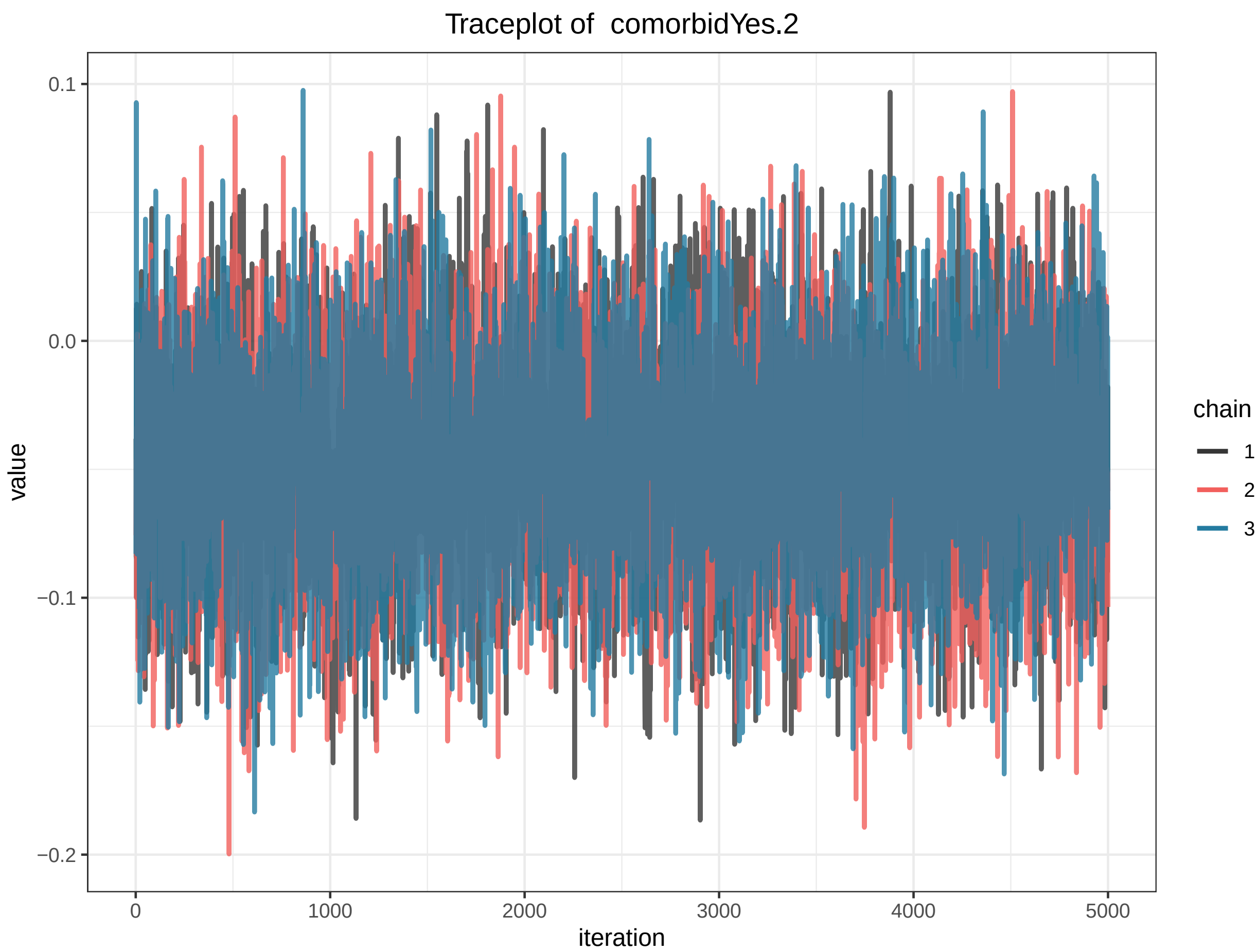

Traceplot of comorbidYes.2
0.1
0.0
−0.1
−0.2
value
0
1000
2000
3000
4000
5000
iteration
chain
1
2
3

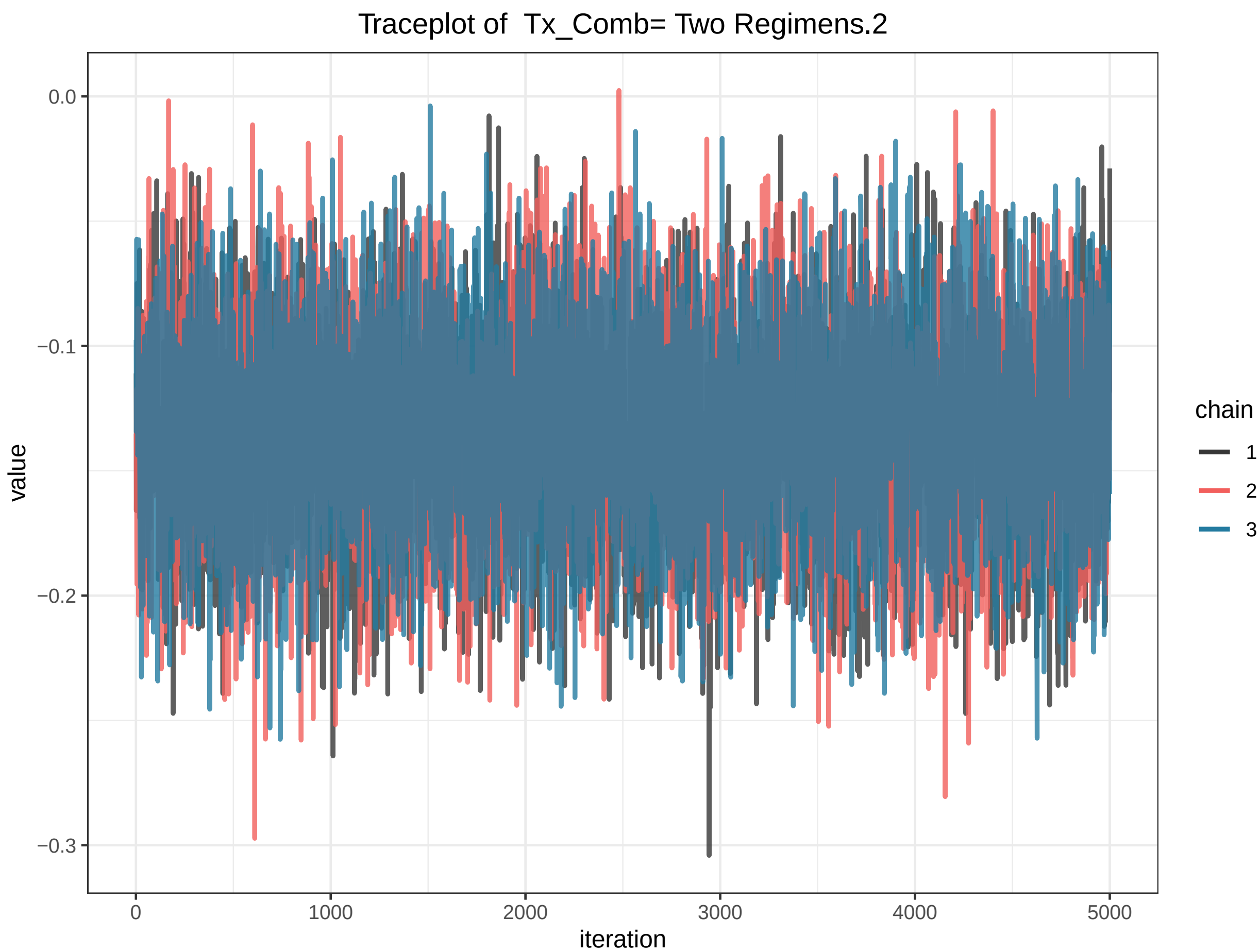
Traceplot of Tx_Comb= Two Regimens.2
value
0.0
−0.1
−0.2
−0.3
iteration
0
1000
2000
3000
4000
5000
chain
1
2
3
