# Supplementary material for: Longitudinal blood pressure trajectories and time to stroke among hypertensive patients: A Bayesian multivariate joint modeling study in Benishangul-Gumuz, Ethiopia
Source: PLoS One. 2026 Oct 23;21(10):e0359435. doi: 10.1371/journal.pone.0359435 (PMC13693927; doi:10.1371/journal.pone.0359435)
Supplement: Supplementary file 4 [file pone.0359435.s005.docx]

**Manuscript ID:** PONE-D-25-65869

**Title:** **Longitudinal Blood Pressure Trajectories and Time to Stroke Among Hypertensive Patients: A Bayesian Multivariate Joint Modeling Study in Benishangul-Gumuz, Ethiopia**

**Response to the Editor**

**Comment :** *Please ensure that your manuscript meets PLOS ONE's style requirements, including those for file naming.*

**Response:** We have revised the manuscript and all associated files to ensure full compliance with the PLOS ONE style requirements and file naming guidelines.

**Comment :** *In the online submission form, you indicated that the datasets used and analyzed during the current study are available from the corresponding author upon reasonable request. All PLOS journals now require all data underlying the findings described in the manuscript to be freely available, unless restricted for ethical or legal reasons.*

**Response:** Thank you for pointing this out. We have revised the Data Availability Statement to comply with the PLOS data-sharing policy. We have uploaded the de-identified dataset as Supporting Information, making the data underlying the findings of this study freely available.

**Comment :** *Please amend the manuscript submission data (via Edit Submission) to include author Helina Mebratu Abegaz.*

**Response:** We have updated the online submission system to include Helina Mebratu Abegaz as a co-author.

**Comment :** *Please ensure that you refer to Figures 4 and 5 in your text as, if accepted, production will need this reference to link the reader to the figure.*

**Response:** We have carefully reviewed the manuscript and ensured that all figures are appropriately cited in the main text.

**Comment :** *Please include captions for your Supporting Information files at the end of your manuscript, and update any in-text citations to match accordingly.*

**Response:** We have added captions for all Supporting Information files at the end of the manuscript and updated the corresponding in-text citations accordingly.

**Comment :** *If the reviewer comments include a recommendation to cite specific previously published works, please review and evaluate these publications to determine whether they are relevant and should be cited. There is no requirement to cite these works unless the editor has indicated otherwise.*

**Response:** We carefully evaluated all recommended references and included those that were relevant and appropriate to our study.

**Response to Reviewer 1**

**Comment:** *Background: The abstract is relatively good, but it should include the study's aim in the background.*

**Response:** Thank you for this helpful suggestion. We have revised the Background section of the abstract to explicitly state the study objective.

**Comment:** *Result: the statement stated as “The incidence of stroke was 2.19 per 100 person-years (95% CI: 1.60–2.64)” is not clear in terms of how many years. It says “2.19 per 100 person-years” does not indicate the number of years.*

**Response:** We respectfully retained the original wording because the incidence rate is correctly expressed as 2.19 per 100 person-years, which is the standard epidemiological measure of incidence density. Person-years represent the cumulative follow-up time contributed by all study participants and therefore do not require specification of a fixed number of follow-up years. This measure appropriately accounts for differences in individual follow-up durations. We believe the current presentation is statistically accurate and consistent with standard reporting practices.

**Comment:** *The first paragraph stated the impact or problem of stroke and hypertension on the global population, Africa, and Ethiopia, which sounds good, but the second paragraph defines hypertension; the order is not good for readers. The definition should come first, followed by the epidemiological impact, or the two paragraphs should be merged.*

**Response:** Thank you for your thoughtful suggestion. We respectfully retained the overall structure of the Introduction because epidemiological manuscripts commonly begin by presenting the public health burden of the disease before introducing its clinical definition. We believe this progression provides an appropriate context for readers. Nevertheless, we carefully revised the Introduction to improve its clarity, coherence, and flow by strengthening the transition between the first and second paragraphs.

**Comment:** *Study Population and Eligibility Criteria: Statements in this section contradict each other and lack clarity. Somewhere it says between January 1, 2015, and September 1, 2025, and later it says between January 1, 2015, and September 1, 2024. Please be clear and consistent throughout the document.*

**Response:** Thank you for identifying this inconsistency. The discrepancy arose from an error during the conversion of dates from the Ethiopian calendar to the Gregorian calendar. We have corrected the dates throughout the manuscript to ensure consistency.The overall study period was January 1, 2015, to December 30, 2024, during which hypertensive patients received follow-up care. Medical records covering this period were retrospectively reviewed, and data extraction was conducted from April 1 to May 10, 2025.The study population consisted of newly diagnosed hypertensive patients aged 18 years or older who initiated follow-up care between January 1, 2015, and September 1, 2024. The September 1, 2024, enrollment cutoff was intentionally selected to ensure that all eligible patients could complete the required minimum of two follow-up visits with documented blood pressure measurements before the end of the study period on December 30, 2024. We have revised the Abstract, Methods, and all other relevant sections to report these dates consistently throughout the manuscript.

**Comment:** *Data collection method: My main doubts are on the source of data, since you are using only secondary data, the majority of pertinent factors like patient behaviors, self-care practices, medication adherence, economy, etc. These factors may influence stroke risk but cannot be obtained from patient charts, which affects the reliability of the study.*

**Response:** We agree that retrospective studies based on routinely collected hospital records are limited by the availability of recorded variables. Information on important factors such as patient behaviours, self-care practices, medication adherence, and socioeconomic status was not consistently available in the medical records and therefore could not be included in the analysis. This limitation is inherent to retrospective chart review studies. However, the use of longitudinal clinical records over an extended follow-up period enabled repeated blood pressure measurements and ascertainment of stroke outcomes, which provided valuable information for addressing the study objective. To better acknowledge this limitation, we have refined the Limitations section of the manuscript to explicitly state that the retrospective design precluded the capture of several potentially important stroke risk factors and that incomplete or inconsistent hospital records may have affected the accuracy of some variables and outcomes.

**Comment:** *The rationale for using Bayesian multivariate joint modeling is appropriate. However, the manuscript does not sufficiently explain why joint modeling provides clinically meaningful advantages over extended Cox models with time-dependent covariates. The explanation remains largely statistical rather than clinical.*

**Response:** Thank you for this constructive comment. We respectfully believe that the rationale for the use of Bayesian multivariate joint modelling is adequately described in the Introduction. Specifically, we explain that previous Ethiopian studies have predominantly used cross-sectional or conventional regression approaches that do not account for repeated blood pressure measurements, within-patient correlation, measurement error, or individual heterogeneity over time. We also highlight that longitudinal blood pressure trajectories provide richer prognostic information than single baseline measurements and that analysing the longitudinal and survival processes separately may result in biased or inefficient estimates. Furthermore, we state that the Bayesian multivariate joint modelling framework simultaneously analyses repeated blood pressure measurements and time-to-stroke outcomes within a unified framework, accounting for measurement error, within-patient correlation, and informative dropout while improving estimation efficiency. To further address the reviewer's concern, we have refined the Introduction to better emphasise the clinical relevance of this approach, namely that longitudinal blood pressure trajectories can improve risk stratification and support earlier identification of hypertensive patients at high risk of stroke.

**Comment:** *There is a serious inconsistency in reported results. In the abstract, SBP and DBP are reported as significant; however, in the Results section, both SBP and DBP are reported as not significant.*

**Response:** We have revised the manuscript to avoid any inconsistency between the Abstract and the main Results section. In both sections, the longitudinal effects of systolic and diastolic blood pressure are reported as statistically significant, with consistent effect estimates and 95% credible intervals. We have now carefully rechecked the entire manuscript and confirmed that the results are reported consistently throughout.

**Comment:** *You calculated a final required sample of 530, but analysed only 494 patients. You mention that 36 incomplete charts were excluded. There is no power recalculation after exclusion and no assessment of selection bias from excluded charts.*

**Response:** The minimum required sample size before accounting for incomplete records was 482 participants. To minimise the impact of potential missing or incomplete data, we added a 10% contingency, resulting in a target sample size of 530. After excluding 36 incomplete medical records, 494 participants remained for analysis, which still exceeded the minimum required sample size of 482. Therefore, the final analytical sample remained adequately powered to address the study objectives, and a post hoc power recalculation was not considered necessary. We have clarified this point in the revised manuscript. We acknowledge that excluding incomplete records may introduce some degree of selection bias, which is an inherent limitation of retrospective chart review studies, and we have further highlighted this in the Limitations section.

**Comment:** *Stroke was defined only as "Documented in the patient's medical chart." This is insufficient. The manuscript does not describe diagnostic criteria (clinical versus imaging), ischaemic versus haemorrhagic classification, ICD codes, or the validation method.*

**Response:** This study was a retrospective chart review conducted in general hospitals using routinely collected clinical records. Stroke cases were identified based on the treating physician's documented diagnosis in the patients' medical records, which reflects routine clinical practice in the participating hospitals. Detailed information on imaging findings, stroke subtype or formal validation procedures was not consistently available in the medical records and therefore could not be incorporated into the study. Similar retrospective studies conducted in comparable resource-limited settings have relied on physician-documented diagnoses obtained from medical records. We acknowledge that this may have resulted in some outcome misclassification and have explicitly included this as a limitation in the revised manuscript.

**Comment:** *You state: "Independence between longitudinal outcomes (SBP and DBP)." This contradicts biological reality. SBP and DBP are strongly correlated. If you used a multivariate joint model, correlation should be modeled.*

**Response:** Thank you for this important comment. We agree that systolic blood pressure (SBP) and diastolic blood pressure (DBP) are biologically correlated. In our Bayesian multivariate joint model, this correlation was explicitly accommodated by jointly modelling the two longitudinal outcomes within a shared modelling framework. The statement referring to "independence" was inaccurate and has been corrected in the revised manuscript.

**Comment:** *You state: "A one-unit increase in systolic blood pressure was linked to a 3.25-fold higher hazard." A one-unit increase (1 mmHg) cannot realistically produce HR = 3.25. Either scaling was applied (e.g., per 10 mmHg), or there is a modelling interpretation error.*

**Response:** Thank you for this important observation. We agree that the original wording was misleading. The reported hazard ratios do not correspond to a 1 mmHg increase in systolic or diastolic blood pressure.Before fitting the longitudinal sub-models, the distributions of systolic blood pressure (SBP) and diastolic blood pressure (DBP) were assessed using histograms and quantile–quantile plots. To improve distributional properties and stabilize the variance, both SBP and DBP were square-root transformed prior to model fitting. Consequently, the association parameters from the Bayesian joint model represent the effect of a one-unit increase in the square-root-transformed underlying blood pressure, rather than a one-unit (1 mmHg) increase in the original measurement scale.To avoid misinterpretation, we have revised the Methods and Results sections to explicitly state that the reported adjusted hazard ratios are interpreted on the square-root-transformed scale and no longer describe them as representing a 1 mmHg increase in blood pressure.

**Comment:** *You report: Median age 55 (IQR 15–18). An IQR cannot be 15–18. This appears to be a typographical or calculation error.*

**Response:** Thank you for identifying this typographical error. We have corrected the value in the revised manuscript. The median age is now reported as 55 years (IQR: 45–60).

**Comment:** *There are frequent grammatical errors and the manuscript requires professional English editing.*

**Response:** We have carefully revised the entire manuscript to improve grammar, sentence structure, clarity, and overall readability. We also corrected typographical errors and ensured consistency throughout the manuscript.

**Overall Minor Comments**

**Comment:** *Figure numbering is inconsistent (Figure 1 repeated).*

**Response:** We have reviewed the manuscript and corrected the figure numbering

**Comment:** *Typographical errors throughout.*

**Response:** We have carefully proofread the manuscript and corrected the identified typographical errors throughout the text.

**Comment:** *The ESC/ESH guideline citation needs updating (consider the 2023 ESC guideline if relevant).*

**Response:** We reviewed the cited guideline and updated the reference where appropriate to ensure that the manuscript reflects the most relevant and current clinical guidance.

**Comment:** *Annex 1 is mentioned but not shown in the text.*

**Response:** Annex 1 has been uploaded as a supplementary file and was available with the revised submission. We have also checked the manuscript and ensured that Annex 1 is appropriately cited and referenced in the relevant section. It is possible that the supplementary file was not displayed in the reviewer view during the initial review process; however, it has now been included and linked appropriately in the revised submission.

**Comment:** *Clarify censoring due to death (competing risk?).*

**Response:** In this study, patients who died before experiencing a stroke were treated as censored observations because information on the specific cause of death was not consistently available in the retrospective medical records.

**Response to Reviewer 2**

**Comment:** *Study Timeline Discrepancy: The abstract (Line 37) states the study period is 2015 to 2025. However, the Methods section (Line 127) specifies that patients were enrolled up to September 1, 2024. This discrepancy makes it unclear whether the data collection is complete, and better to fix it.*

**Response**: Thank you for identifying this inconsistency. The discrepancy resulted from an error during the conversion of dates from the Ethiopian calendar to the Gregorian calendar. We have corrected the study period throughout the manuscript to ensure consistency. The correct study period was January 1, 2015, to December 30, 2024, during which hypertensive patients received follow-up care. Medical records covering this period were retrospectively reviewed, and data extraction was conducted from April 1 to May 10, 2025. Eligible participants were newly diagnosed hypertensive patients aged 18 years or older who initiated follow-up care between January 1, 2015, and September 1, 2024. The enrollment cutoff of September 1, 2024, was intentionally selected to ensure that all included patients had sufficient time to complete the required minimum of two follow-up visits with documented blood pressure measurements before the end of the study period on December 30, 2024. The Abstract, Methods, and all other relevant sections have been revised to report these dates consistently throughout the manuscript.

**Comment:** *Statistical Clarity: In the Abstract results, you report AHR (Adjusted Hazard Ratio) with 95% CI. Since the researchers are using a Bayesian model, ensure that these are clearly defined as posterior summaries (e.g., posterior mean/median etc.).*

**Response:** Thank you for this valuable suggestion. We agree that Bayesian analyses should be clearly identified. In the revised abstract, we have clarified that the reported hazard ratios are accompanied by 95% credible intervals (CrI. We retained the term adjusted hazard ratio (AHR) because the estimates are adjusted for the covariates included in the multivariable Bayesian joint model. Detailed model specifications, including the Bayesian estimation procedure and posterior inference, are provided in the Methods section and supplementary file 1.

**Comment:** *The intro provides a strong epidemiological funnel. However, the projection that hypertension will rise to 125.5 million by 2025 (Line 68) should be updated to reflect that 2025 has passed; please adjust the tense to indicate if this threshold has already been surpassed.*

**Response:** We have revised the sentence accordingly to reflect that the projection referred to an estimate while preserving the original meaning of the cited source.

**Comment:** *Major Chronological Error: There is a contradiction in the data extraction timeline. The study period is listed through September 2025, whereas data extraction is reported to have occurred between April 1 and May 10, 2025. It is chronologically impossible to extract data in April/May 2025 that tracks patient outcomes through September 2025. Please harmonize these dates immediately.*

**Response:** Thank you for bringing this to our attention. We agree that the originally reported dates were inconsistent. The correct study period was January 1, 2015, to December 30, 2024. Accordingly, all patient follow-up and outcome ascertainment were completed by December 30, 2024. Medical records from this period were retrospectively reviewed, and data extraction was subsequently conducted between April 1 and May 10, 2025, which is chronologically consistent with the study design. To ensure that all participants had at least two documented follow-up visits with blood pressure measurements before the end of follow-up, only newly diagnosed hypertensive patients who initiated follow-up care between January 1, 2015, and September 1, 2024 were eligible for inclusion. We have corrected the study period in the Abstract, Methods, and all other relevant sections of the manuscript to ensure that the dates are reported consistently throughout.

**Comment:** *Please verify that the equation typesetting contains duplicate indices, which is mathematically ambiguous. The notation in Equation (1) should clearly distinguish between observed measurements and true trajectories (*$m_{i}\left( t \right)$ *vs.* $y_{ki}\left( t \right)$*).*

**Response:** We have now carefully reviewed and revised the statistical model section to improve the mathematical notation and ensure clarity. Specifically, we have revised Equation (1) to clearly distinguish between the observed longitudinal measurements $y_{ki}\left( t \right)$) and the underlying true longitudinal trajectories (($m_{i}\left( t \right)$)). We have also corrected the indexing notation to avoid ambiguity and ensure consistency throughout the longitudinal sub-model, survival sub-model, and Bayesian multivariate joint model formulation.

**Comment:** *Statistical Values: In the result tables, there is a recurring tendency to label Bayesian posterior estimates under standard "CHR/AHR (95% CI)" columns. Please ensure all Bayesian estimates (AHR) are accompanied by CrI (Credible Intervals), not CI (Confidence Intervals).*

**Response:** Thank you for this important observation. We have carefully reviewed all result tables and revised the column headings and statistical terminology accordingly. All Bayesian posterior hazard ratio estimates are now consistently reported as adjusted hazard ratios (AHRs) with 95% credible intervals (CrI) rather than confidence intervals (CI).

**Comment:** *Check for empty cells or identical values (e.g., Mean (-0.02, -0.02)) in Table 3. Such values suggest potential model convergence issues or printing errors.*

**Response:** The apparent identical values (e.g., −0.02, −0.02) were due to rounding very small regression coefficients and their corresponding 95% credible intervals to two decimal places, rather than model convergence or estimation problems. To avoid this ambiguity, we have revised Table 3 by reporting these estimates to three decimal places where appropriate. This more accurately reflects the posterior estimates and their 95% credible intervals and prevents the appearance of identical lower and upper limits. We also rechecked the model output to ensure that all reported values are consistent with the final fitted Bayesian joint model.

**Comment:** *Variable Scaling: Ensure that researchers explicitly state if blood pressure measurements were log-transformed for the joint model, as the intercept fixed effect for SBP (12.23) is much lower than standard clinical mmHg readings.*

**Response:** We clarify that blood pressure measurements were not log-transformed in the joint model. Instead, systolic blood pressure (SBP) and diastolic blood pressure (DBP) were square-root transformed before fitting the longitudinal mixed-effects sub-models to improve distributional properties and stabilize the variance. Consequently, the intercepts and regression coefficients from the longitudinal sub-model are reported on the square-root-transformed scale rather than the original mmHg scale. The survival sub-model estimates, including the association parameters and adjusted hazard ratios, remain interpretable on the hazard scale. To avoid ambiguity, we have revised the Methods section to explicitly describe the transformation and clarify the interpretation of the longitudinal model estimates.

**Comments:** *Urban-Rural Interpretation: The discussion and conclusion sections contain a contradiction. Your results (Line 423) suggest that rural residence is protective against stroke, yet your conclusion (Line 429) states that interventions should prioritize urban populations to reduce the burden. If rural residence is protective, the recommendation should be to investigate the factors driving this protection in rural areas rather than prioritizing urban centers as a direct stroke-reduction intervention, unless the high-risk group is specifically located there. Please reconcile this logic.*

**Response:** We agree that the conclusion should be fully consistent with the study findings. In the revised manuscript, we have corrected this inconsistency by recommending targeted stroke prevention strategies for urban residents, who were at higher risk of stroke in our study, while also emphasizing the need for future research to investigate the contextual and healthcare-related factors that may underlie the observed lower stroke risk among rural residents. The conclusion has been revised accordingly.
