# Supplementary material for: Longitudinal blood pressure trajectories and time to stroke among hypertensive patients: A Bayesian multivariate joint modeling study in Benishangul-Gumuz, Ethiopia
Source: PLoS One. 2026 Oct 23;21(10):e0359435. doi: 10.1371/journal.pone.0359435 (PMC13693927; doi:10.1371/journal.pone.0359435)
Supplement: Supplementary file 5 [file pone.0359435.s003.docx]

**Reviewing the research article**

Title: “Longitudinal Blood Pressure Trajectories and Time to Stroke Among Hypertensive Patients: A Bayesian Multivariate Joint Modeling Study in Benishangul-Gumuz, Ethiopia”

**Abstract**

**Background**: The abstract is relatively good, but it should include the study's aim in the background.

**Result**: the statement stated as “The incidence of stroke was 2.19 per 100 person-years (95% CI: 1.60–2.64)” is not clear in terms of how many years. It says “2.19 per 100 person-years” does not indicate the number of years.

**Introduction**

The first paragraph stated the impact or problem of stroke and hypertension on the global population, Africa, and Ethiopia, which sounds good, but the 2^nd^ paragraph defines hypertension; the order is not good for readers 1^st^ definition 2^nd^ epidemiological impact of the problem. So, please rewrite the 2^nd^ paragraph or merge it with the 1^st^ paragraph.

**Methods**

Study Population and Eligibility Criteria:

Statements in this sections contradicts each other and lack clarity

Somewhere it says between January 1, 2015, and September 1, 2025, at the general hospitals of Benishangul Gumuz, and later it says between January 1, 2015, and September 1, 2024”. So, which one is correct? Be clear and consistent throughout the document.

Data collection method: My main doubts are on the source of data, since you are using only secondary data, the majority of pertinent factors like patient behaviors, self-care practices, medication adherence, economy….. This exposes patients to a stroke that cannot be availed at charts, so this affects the reliability of the study.

Major comments to be addressed

- The rationale for using Bayesian multivariate joint modeling is appropriate. However:
- The manuscript does not sufficiently explain why joint modeling provides clinically meaningful advantages over extended Cox models with time-dependent covariates.
- The explanation remains largely statistical rather than clinical.
- There is a serious inconsistency in reported results:
- In the abstract:
- SBP AHR = 3.25 (95% CrI: 1.85–5.83) and DBP AHR = 2.32 (95% CrI: 1.35–4.27) say significant; however, in the result section, both SBP and DBP say **NOT significant**.
- You calculated a final required sample of 530, but analyzed only 494 patients.
- You mention that 36 incomplete charts were excluded.
- There is no power recalculation after exclusion.
- No assessment of selection bias from excluded charts.
- Stroke was defined only as:

“Documented in the patient’s medical chart.” This is insufficient. Which does not incorporate:

- Diagnostic criteria (clinical vs imaging), ischemic vs hemorrhagic classification, ICD codes? And the validation method?
- You state:

“Independence between longitudinal outcomes (SBP and DBP).”

This contradicts biological reality. SBP and DBP are strongly correlated.

If you used a multivariate joint model, correlation should be modeled.

- You state:

“A one-unit increase in systolic blood pressure was linked to a 3.25-fold higher hazard.”

A one-unit increase (1 mmHg) cannot realistically produce HR=3.25.

This suggests:

- Either scaling was applied (e.g., per 10 mmHg), OR
- There is a modeling interpretation error.
- You report:

Median age 55 (IQR 15–18)

An IQR cannot be 15–18. This appears to be a typographical or calculation error.

- There are frequent grammatical errors:

Examples:

- “Trajectories of were first modeled”
- “Who was enrolled”
- “Were classified based on The 2018 ESC”
- Repeated figure numbering inconsistencies

The manuscript requires professional English editing.

**Overall minor comments**

- Figure numbering is inconsistent (Figure 1 repeated).
- Typographical errors throughout.
- ESC/ESH guideline citation needs updating (consider 2023 ESC if relevant).
- Annex 1 is mentioned but not shown in the text.
- Clarify censoring due to death (competing risk?).

**Summary**

This manuscript addresses an important public health issue in Ethiopia and other low-income countries: the dynamic relationship between longitudinal changes in blood pressure (BP) and time to stroke. The use of Bayesian multivariate joint modeling represents a methodological strength and demonstrates advanced statistical application. The study is relevant, potentially impactful, and suitable for submission to a journal such as *PLOS ONE*
