# Supplementary material for: Longitudinal blood pressure trajectories and time to stroke among hypertensive patients: A Bayesian multivariate joint modeling study in Benishangul-Gumuz, Ethiopia
Source: PLoS One. 2026 Oct 23;21(10):e0359435. doi: 10.1371/journal.pone.0359435 (PMC13693927; doi:10.1371/journal.pone.0359435)
Supplement: Supplementary file 6 [file pone.0359435.s004.docx]

**A Point-by-point Response**

As an independent reviewer, I have evaluated the manuscript **"Longitudinal Blood Pressure Trajectories and Time to Stroke Among Hypertensive Patients: A Bayesian Multivariate Joint Modeling Study in Benishangul-Gumuz, Ethiopia"** (PONE-D-25-65869).

The study employs an advanced statistical approach to address the critical public health challenge of stroke risk in hypertensive patients. While the methodological rigor is commendable, several significant discrepancies regarding study timelines and data transparency must be addressed before the manuscript can be considered for publication.

Below is the detailed, point-by-point review.

**1. Abstract and Introduction**

- **Study Timeline Discrepancy:** The abstract (Line 37) states the study period is **2015 to 2025**. However, the Methods section (Line 127) specifies that patients were enrolled up to **September 1, 2024**. This discrepancy makes it unclear whether the data collection is complete, and better to fix it.
- **Statistical Clarity:** In the Abstract results, you report **AHR** (Adjusted Hazard Ratio) with **95% CI**. Since the researchers are using a Bayesian model, ensure that these are clearly defined as posterior summaries (e.g., posterior mean/median etc).
- **Introduction Refinement:** The intro provides a strong epidemiological funnel. However, the projection that hypertension will rise to 125.5 million by 2025 (Line 68) should be updated to reflect that 2025 has passed; please adjust the tense to indicate if this threshold has already been surpassed.

**2. Methods and Materials**

- **Major Chronological Error:** There is a contradiction in data extraction timeline:
  - Line 112: The study period is listed through **September 1, 2025**.
  - Line 113: state data extraction occurred between **April 1 and May 10, 2025**.

It is chronologically impossible to extract data in April/May 2025 that tracks patient outcomes through September 2025. Please harmonize these dates immediately.

- **Mathematical Notation Issues:** Please verify that the equation typesetting contains duplicate indices, which is mathematically ambiguous. The notation in Equation (1) should clearly distinguish between observed measurements and true trajectories ($m_{i}\left( t \right)$ vs. $y_{ki}\left( t \right)$).

**3. Results**

- **Statistical Values**: In the result tables, there is a recurring tendency to label Bayesian posterior estimates under standard "CHR/AHR (95% CI)" columns. Please ensure all Bayesian estimates (AHR) are accompanied by **CrI** (Credible Intervals), not CI (Confidence Intervals).
- Check for empty cells or identical values (e.g., Mean (-0.02, -0.02)) in Table 3. Such values suggest potential model convergence issues or printing errors.
- **Variable Scaling:** Ensure that researchers explicitly state if blood pressure measurements were log-transformed for the joint model, as the intercept fixed effect for SBP (12.23) is much lower than standard clinical mmHg readings.

**4. Discussion and Conclusions**

- Urban-Rural Interpretation: The discussion and conclusion sections contain a contradiction. Your results (Line 423) suggest that rural residence is protective against stroke, yet your conclusion (Line 429) states that interventions should prioritize urban populations to reduce the burden. If rural residence is protective, the recommendation should be to investigate the factors driving this protection in rural areas rather than prioritizing urban centers as a direct stroke-reduction intervention, unless the high-risk group is specifically located there. Please reconcile this logic.

**Conclusion**

The manuscript utilizes a high-level statistical framework (Bayesian multivariate joint modeling) that is highly relevant for medical journals. However, the internal contradictions regarding the study dates, the typographical errors in the result tables, and the conflicting conclusions preclude acceptance in the current state.
